# Supramolecular Assembly and Thermogelation Strategies Using Peptide–Polymer Conjugates

**DOI:** 10.1021/acs.biomac.4c00031

**Published:** 2024-04-25

**Authors:** Chloé Pascouau, Maren Schweitzer, Pol Besenius

**Affiliations:** Department of Chemistry, Johannes Gutenberg-University Mainz, Duesbergweg 1014, D-55128 Mainz, Germany

## Abstract

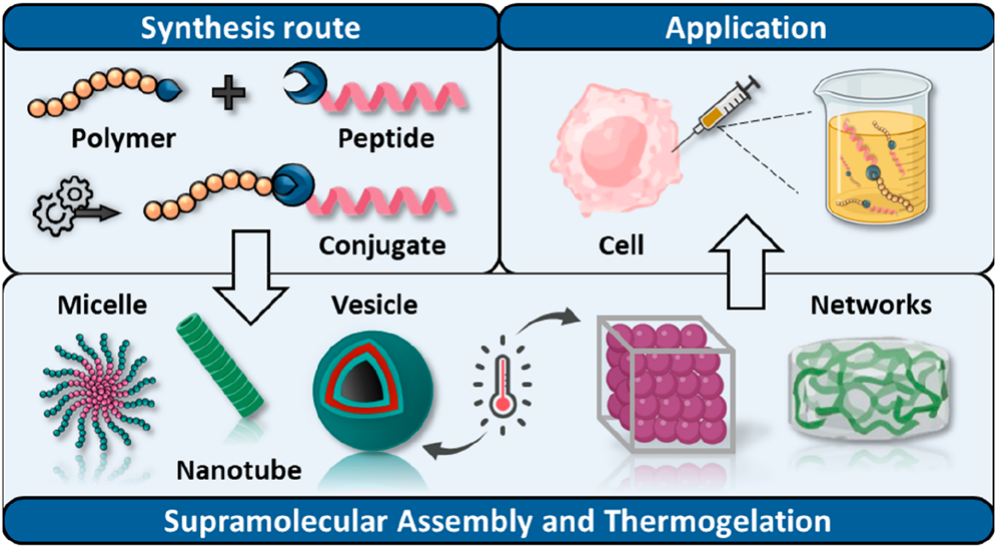

Peptide–polymer conjugates (PPCs) are of particular interest in the development of responsive, adaptive, and interactive materials due to the benefits offered by combining both building blocks and components. This review presents pioneering work as well as recent advances in the design of peptide–polymer conjugates, with a specific focus on their thermoresponsive behavior. This unique class of materials has shown great promise in the development of supramolecular structures with physicochemical properties that are modulated using soft and biorthogonal external stimuli. The temperature-induced self-assembly of PPCs into various supramolecular architectures, gelation processes, and tuning of accessible processing parameters to biologically relevant temperature windows are described. The discussion covers the chemical design of the conjugates, the supramolecular driving forces involved, and the mutual influence of the polymer and peptide segments. Additionally, some selected examples for potential biomedical applications of thermoresponsive PPCs in tissue engineering, delivery systems, tumor therapy, and biosensing are highlighted, as well as perspectives on future challenges.

## 1 Introduction

In recent years, the scientific community has faced challenges in the design of materials with new properties and functionalities. One promising approach is to conjugate natural or naturally inspired molecules with other entities, such as synthetic polymers, to combine the benefits of the building blocks.^1,2^ The ability to conjugate a polymer with a biomolecule is a concept that emerged in the 1970s.^3^ Since the synthesis of the first protein–polymer conjugate in 1977 by Abuchowski et al.,^4^ conjugates have gained increasing interest. In particular, peptide-based building blocks have been extensively studied thanks to their ability to interact through inter- and intramolecular interactions, allowing the formation of well-defined secondary structures in aqueous media.^5^ Moreover, due to the use of peptide synthesis on solid supports, the synthesis of peptides with precise sequences is an easily achievable process that can be performed on a larger scale.^6^

Advances in polymer synthesis have enabled the development of controlled polymerization techniques for the design of well-defined polymers with diverse and tunable architectures.^7−9^ When combined with an understanding of the structures and functions of biomolecules, PPCs represent a unique class of (bio)macromolecules for the development of responsive and interactive materials. The conjugation of polymers to biomolecules can enhance solubility and stability while also combining the benefits of polymer scalability and peptide structural diversity.^10^ As a result, conjugates can be applied in various applications including biomedical areas (tissue engineering, delivery systems), nano- and biotechnology.^11,12^ Thanks to the wide variety of polymers and amino acids, whether natural or synthetic, the versatility of the self-assembled structures and the range of properties of these materials can be adjusted by introducing diverse chemical functionalities.^13^ The possibility to perform on-demand control of the material’s properties is particularly appealing for targeted applications.^14^ By introducing a stimuli-responsive building block,^15^ external stimuli such as temperature, pH value, redox potential, light or solvent composition can trigger responses in the supramolecular assembly based on the polymer or peptide structure.^16−19^ This offers unique opportunities to tune the parameter space required for the design of advanced (bio)materials by using thermal, chemical, mechanical or biochemical cues.

Although various stimuli-responsive PPCs have been discussed in the literature, the focus of this review is dedicated to temperature as an input signal. If used as a turn-on signal in a biologically relevant temperature window, this external stimulus is particularly appealing for biomaterial design purposes for structuring and processing cellular scaffold materials or injectable biopharmaceuticals. Here, we discuss pioneering work and advances on synthetic peptide–polymer conjugates, along with recent strategies dedicated to the development of thermal protocols to trigger, direct, and fix supramolecular assemblies. The provided examples cover conjugates that display temperature-induced self-assembly in dilute solution or gelation at a higher solid-weight content. We describe conjugates consisting of both peptide and polymer segments of synthetic or natural origin. Thus, the synthetic routes, design, and characterization of various thermoresponsive PPCs are presented first (section 2), followed by their stepwise supramolecular assembly into well-defined structures (section 3) and gelation (section 4) as a function of temperature. The discussion covers the peptide-driven self-assembly and the influence of the polymer on the formation of structures with different dimensions, including 0D nanospheres and nanoparticles, 1D nanotubes and nanofibers, 2D nanosheets and nanoribbons, and 3D networks. Additionally, we highlight potential biomedical applications of temperature-responsive peptide–polymer conjugates in delivery applications, tissue engineering, and biosensing systems in section 5.

## 2 Design of Thermoresponsive Peptide–Polymer Conjugates

In the past decades, numerous methodologies have been reported for the development of synthetic approaches to design peptide- or protein-based polymer conjugates. Advances in controlled polymerization techniques, such as reversible addition–fragmentation by chain transfer polymerization (RAFT),^20^ atom transfer radical polymerization (ATRP),^9^ nitroxide-mediated radical polymerization (NMP),^21^ and ring-opening polymerization (ROP),^22^ have enabled the synthesis of well-defined architectures of synthetic polymers. The preparation of PPCs involves three main categories of synthetic techniques: grafting to, grafting from, and grafting through strategies. The grafting approach is a straightforward method widely employed for conjugating biomolecules. Here, the peptide and polymer are prepared separately and coupled in a second step, usually after the purification of the individual building blocks. In the grafting from strategy, the peptide either initiates the polymerization or the synthetic polymer initiates the synthesis of the biomolecule. In other words, the polymer is synthesized from the biomolecule or the biomolecule is synthesized from the polymer. In the case of the grafting through approach, the polymerization is achieved with a biomolecule that bears a polymerizable group, acting as a macromonomer. We refer the interested reader to existing reviews that cover synthetic routes for the preparation of PPCs.^23−28^

Thermoresponsive PPCs have been designed using the grafting to, grafting from, and grafting through strategies based on coupling or polymerization techniques.^29^ In the grafting to approach, the polymer and the biomolecule are conjugated by coupling reactions through biorecognition or covalent reactions, including 1,3-dipolar cycloaddition chemistry, thiol-maleimide addition, and others. In the case of the grafting from and grafting through methods, the synthesis of the conjugates occurs via polymerization conjugation. Indeed, the peptide fragment can be used as a macroinitiator for polymerization techniques, including ATRP, single-electron transfer living radical polymerization (SET-LRP), or NMP. The peptide can also be employed as a macromonomer for radical polymerization or as a chain transfer reagent (CTA) to perform RAFT and radical polymerization. Another conjugation by polymerization involves the ROP of *N*-carboxyanhydrides (NCA) using a polymer as a macroinitiator. In recent years, a wide range of thermoresponsive PPCs have been developed.^30,31^ This second section provides an overview of the design, synthesis, and characterization of temperature-responsive PPCs using a variety of peptide systems and architectures.

### 2.1 ELP-Based Polymer Conjugates

Among the polypeptide family, the well-studied elastin-like polypeptides (ELPs) are biopolymers derived from the elastin protein that exhibit lower critical solution temperatures that operate in a biologically relevant temperature window.^32^ The particular sequence of ELPs consists of the amino acid repeat V-P-G-X-G for valine, proline, and glycine, with X any amino acid excluding P. Due to the tunable temperature-dependent properties of ELPs, these peptides have been the subject of numerous studies over the years for the development of thermoresponsive bioconjugates.^33,34^

**Figure 1:**
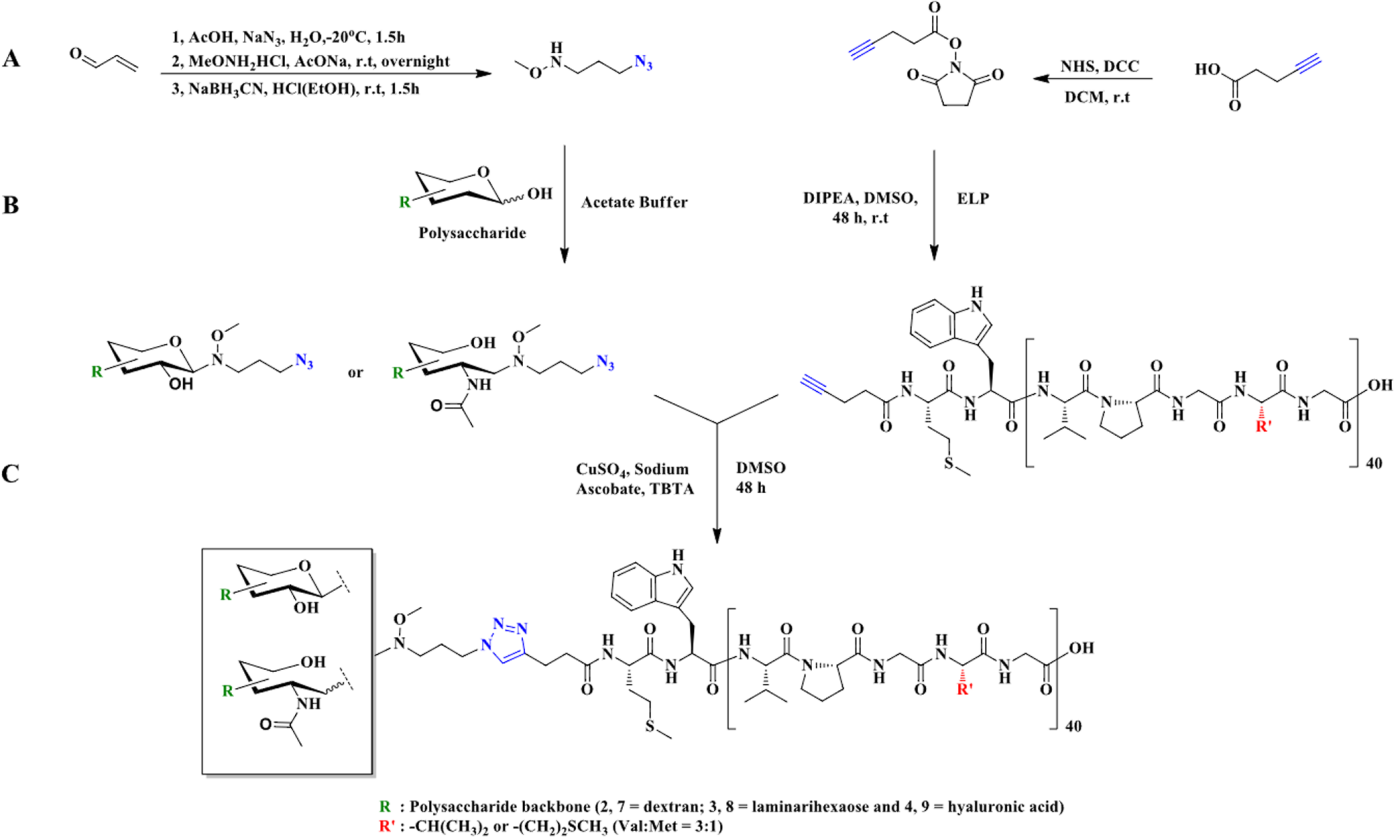
Synthesis route of the ELP-based polysaccharide conjugates. (A) Bifunctional linker and NHS-alkyne ester synthesis. (B) Functionalization and modification of polysaccharides and ELPs. (C) Coupling of the ELPs and polysaccharides. Adapted with permission from ref (37). Copyright 2020 American Chemical Society.

ELP conjugates were synthesized by ring-opening polymerization (ROP) of γ-benzyl-l-glutamate *N*-carboxyanhydride using ELP as macroinitiator in the team of Lecommandoux.^35^ Several poly(l-glutamic acid)-*block*-elastin-like polypeptides (E_*n*_-*b*-ELP) with various chain lengths of the (E)_*n*_ block were obtained. The corresponding diblock copolymers showed molar masses *M̅*_*n*_ between 26 500 and 32 500 g/mol with narrow dispersities *Đ* = 1.02–1.06, supporting a well-controlled polymerization. In a recent study, the team developed an ELP-based ABC triblock copolymer for hydrogel formation.^36^ The copolymer was composed of a first hydrophobic poly(trimethylene carbonate) (PTMC) block (A) and a hydrophilic–hydrophobic recombinant ELPs diblock (BC), with block C being thermoresponsive. The ring-opening polymerization of trimethylene carbonate (TMC) was performed for the synthesis of the first block. [4-(Azidomethyl)phenyl]methanol was used as the initiator for the ROP of TMC, installing an azide group at the chain end of the PTMC (PTMC-N_3_) for coupling reaction. In parallel, two chemoselective modification steps of the following ELP diblock MW[VPGVGVPGMG(VPGVG)_2_]_15_[VPGIG]_20_C allowed to obtain the alkyne-terminated BC diblock, where M, W, V, P, G, C, and I represent the one-letter amino codes for methionine, tryptophan, valine, proline, glycine, and isoleucine. The PTMC-N_3_ (block A) and ELP (diblock BC) were finally coupled by click-chemistry through copper-catalyzed azide–alkyne cycloaddition (CuAAC), allowing the formation of the copolymer PTMC_30_-*b*-MW-(ELP[V_3_M_1_-60])-(ELP[I_1_-20])-C(N-EtSucc), using the Chilkoti nomenclature. ELPs were also used in the design of temperature-responsive PPCs with polysaccharide blocks.^37^ To synthesize the bioconjugates, various polysaccharides, including hyaluronic acid (HA), laminarihexaose (Lam), and dextran (Dex), were employed. Alkyne-functionalized ELP was first prepared for subsequent coupling to the polysaccharide. For this purpose, the N-terminus of ELP was coupled with an alkyne-functionalized *N*-hydroxysuccinimide ester (NHS-ester) (Figure 1A and 1B). Then, a bifunctional linker consisting of azide and *N*-methoxyamine groups was utilized for the modification of the polysaccharides (Figure 1A and 1B). By conjugation of the *N*-methoxyamine group to the reducing end of the polysaccharides, azide-functionalized polysaccharides were obtained. Finally, a CuAAC reaction was performed using the modified ELP and polysaccharide blocks, yielding three different bioconjugates of the type ELP-*b*-polysaccharide (Figure 1C). Further investigations were reported describing the synthesis of a variety of ELP-*b*-HA bioconjugates.^38^ By variation of the chain length and composition, nine ELP-*b*-HA derivatives were prepared using a similar synthetic approach. In this case, the ELP and HA blocks were coupled by a strain-promoted azide–alkyne cycloaddition (SPAAC) reaction.

### 2.2 Collagen-Based Polymer Conjugates

Collagen mimetic peptides (CMPs) derived from natural collagen are another extensively studied group of tunable synthetic peptides.^39^ Collagen is defined by a specific amino acid sequence, X-Y-G, where the Y and X amino acids are generally hydroxyproline (O) and proline (P).^40^ The structural specificity of collagen lies in the formation of a triple helical domain. Collagen has been employed in the development of thermoresponsive peptide conjugates.^41^ Krishna et al. designed a conjugate consisting of a collagen mimetic peptide and poly(diethylene glycol methyl ether methacrylate) (PDEGMEMA).^42^ The temperature-responsive PDEGMEMA was synthesized by RAFT polymerization. The use of a functional chain transfer agent (CTA) enabled the functionalization of one chain end of PDEGMEMA with an active ester. Further coupling was carried out using a collagen mimetic peptide functionalized with an amine group at both chain ends. The reaction between the active ester and amine groups resulted in the formation of a coil–rod–coil triblock conjugate. Static light scattering (SLS) measurements revealed an LCST transition of the conjugate at approximately 35 °C. In a more recent study, the group reported diblock conjugates using PDEGMEMA and collagen mimetic peptide,^43^ using a similar synthetic route based on coupling an amine with an active ester. Recently, recombinant human collagen (RHC) and polysaccharide conjugates have been developed for the synthesis of thermoresponsive and biocompatible cross-linked hydrogels.^44^ To this end, the carboxylic acids of RHC were first activated using 1-ethyl-3-(3-(dimethylamino)propyl)carbodiimide (EDC) and further reacted with *N*-hydroxysuccinimide (NHS) to yield the NHS ester derivatives. To support conjugate solubility and efficient conjugation, higher equivalents of NHS compared to EDC were used. Finally, after the addition of chitosan, the bioconjugate was obtained by reaction with the amine groups of chitosan. Furthermore, the synthesis of temperature-responsive conjugates based on collagen and ELPs has also been extensively reported by the Kiick group.^45−47^

### 2.3 Amyloid-β-Based Polymer Conjugates

The ability of peptides to form fibrils or ribbons through β-sheet secondary structure is well-documented and was one of the reasons for intense investigations using, for example, the amyloid-β peptide sequences.^48^ Aggregation of the amyloid-β peptide into fibrils has notably received considerable attention due to its role in several neurodegenerative diseases, including Alzheimer’s disease.^49^ A series of investigations using polymer conjugates of full length amyloid-β_1–40_ were reported^23^ or shorter peptide sequences like amyloid-β_17–20_, LVFF for leucine, valine, and phenylalanine, which are very hydrophobic and have a strong driving force for fibrillation.^50^

**Figure 2:**
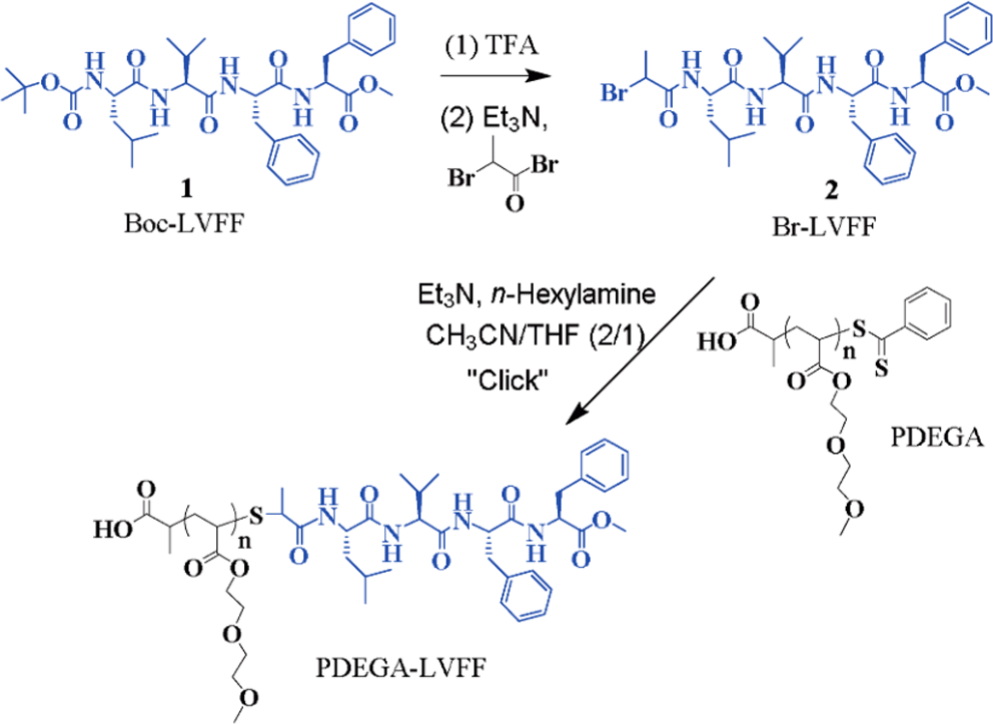
Synthetic route for PPCs consisting of poly(diethylene glycol methyl ether acrylate) (PDEGA) and a short amyloid-β_17–20_ LVFF peptide segment. Adapted with permission from ref (51). Copyright 2017 John Wiley and Sons.

Thermoresponsive PPCs were designed for example in the Binder group through a combination of aminolysis and thio-bromo “click” reactions.^51^ The conjugate consisted of a polymer block of poly(diethylene glycol methyl ether acrylate) (PDEGA) and a short amyloid-β_17–20_ LVFF peptide segment. The PDEGA polymer was synthesized by RAFT polymerization of diethylene glycol methyl ether acrylate (DEGA) using azobis(isobutyronitrile) (AIBN) as the initiator and 2-(phenylcarbonothioylthio)propanoic acid as the CTA. The LVFF sequence of the amyloid-β_17–20_ peptide was synthesized by solution-phase peptide synthesis, followed by subsequent deprotection to form the H_2_N–LVFF–OMe peptide. An amidation reaction was used to introduce the electrophilic 2-bromoacetamide group to the N-terminus of the peptide. Aminolysis of the dithioester to a thiol end-group on the PDEGA polymers, followed by nucleophilic substitution of the bromo-functionalized LVFF peptide, yielded the final PPCs via a thio-bromo “click” reaction (Figure 2), with various chain lengths ranging from *M̅*_*n*_ = 5 600 to 8900 g/mol and low dispersities of *Đ* = 1.21–1.15. Analysis of the thermoresponsive properties of the PPC showed an LCST transition at a temperature of around 44.7 °C. The introduction of the hydrophobic peptide on the polymer decreased its hydrophilicity, resulting in a decrease in the LCST of the initial PDEGA (54.8 °C).

In another study, the same short amyloid-β peptide segment was used as a CTA in the design of temperature-responsive PPCs.^52^ The LVFF amyloid-β_17–20_ peptide sequence was synthesized by solution-phase peptide synthesis and coupled with a RAFT-based agent to form the peptide-functionalized CTA. RAFT polymerization of di(ethylene glycol) methyl ether methacrylate (DEGMA) was performed, resulting in amyloid-β-peptide-based polymer conjugates exhibiting LCST transitions at around 26 °C.

### 2.4 Cyclic Peptide-Based Polymer Conjugates

Cyclic peptides consisting of 4–12 motifs with an alternating sequence of l- and d-amino acids, have received increased interest due to their ability to self-assemble into nanotubes, which can potentially be used in membrane channeling.^53,54^ In recent years, a wide range of PPCs containing cyclic peptides has been developed^55−57^ using the robust assembly motif and have been extensively explored in PPC design.

**Figure 3:**
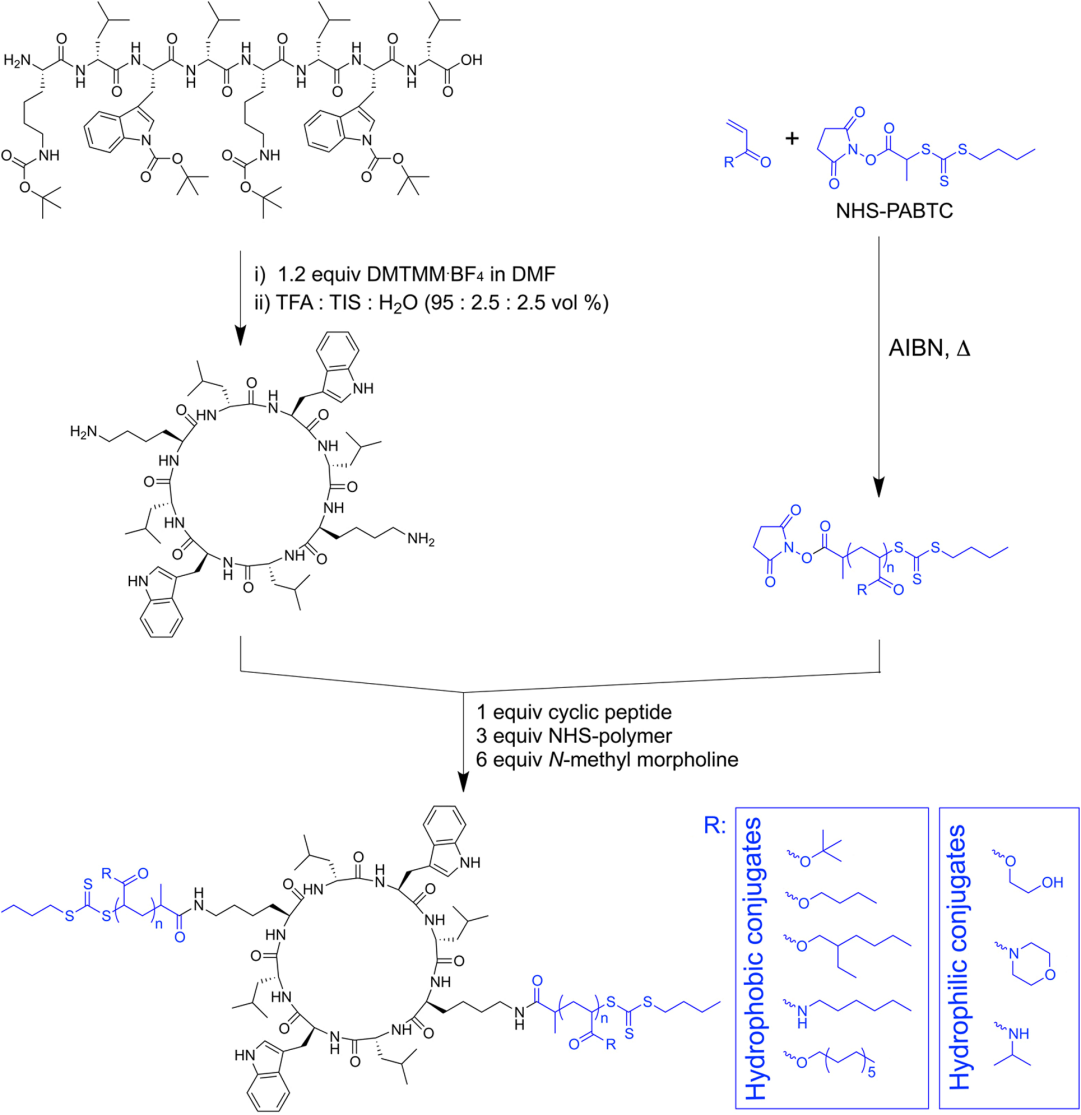
Synthesis route for cyclic peptide–polymer conjugates. Adapted with permission from ref (59). Copyright 2014 American Chemical Society.

Chapman et al. investigated the development of thermoresponsive cyclic peptide–polymer conjugates.^58^ For instance, the conjugation of poly(2-ethyl-2-oxazoline) (pEtOx) to a cyclic octapeptide was performed by “click” chemistry. The temperature-responsive pEtOx polymers were synthesized by cationic ROP by using an alkyne-functionalized initiator. The resulting polymers were obtained with degrees of polymerization (DP) of 20 and 40 and displayed narrow dispersities *Đ* ≤ 1.20. The cyclic peptide was designed by a structure composed of 8 amino acids with alternating l- and d-chirality, bearing two azide functionalities. Conjugates with different DPs were obtained by CuAAC coupling of the cyclic peptide to two pEtOx chains. In contrast to the conjugate bearing pEtOx chains with DP = 20, which showed limited solubility in H_2_O, the cyclic peptide containing the pEtOx chains with a DP = 40 ((pEtOx_40_)_2_–CP) exhibited a LCST transition at around 70 °C due to its longer chain length. In a related study, the team employed a similar cyclic peptide but conjugated to either hydrophilic or hydrophobic polymers.^59^ The peptide sequence was initially synthesized by using solid phase peptide synthesis (SPPS) and subsequently cyclized (Figure 3). RAFT polymerization was performed to synthesize all polymer derivates. An NHS-based chain transfer agent was used to afford polymers with an NHS ester at the chain end. By reacting the two lysines of the cyclic peptide with the active ester located at the polymers’ chain ends, a series of cyclic peptide–polymer conjugates was obtained.

### 2.5 Polypeptide-Based Polymer Conjugates

Over the past decades, the synthesis of polypeptides by ROP of *N*-carboxyanhydride (NCA) has been the subject of extensive research.^60,61^ Diverse properties and architectures of polypeptides have been developed by combining their ability to form secondary structures with scalable and controlled polymerization methods. Conjugation of synthetic polypeptides with a range of other polymers has enabled their use in the development of thermoresponsive conjugates.^62−64^

For instance, a temperature-responsive PPC consisting of polytyrosine (Y)_*n*_ and PEtOx was synthesized by a “grafting onto” method.^65^ Initially, the authors conducted the cationic ROP of 2-ethyl-2-oxazoline and chemically modified the chain end using NaN_3_ to form an azide-functionalized PEtOx block. In parallel, the propargyl-functionalized polytyrosine block was synthesized by ROP of NCA of tyrosine, followed by a postmodification through etherification with propargyl bromide. The two blocks were finally coupled by CuAAC. Amphiphilic grafted Y_*n*_-*g*-PEtOx copolymers with various pendant and backbone chain lengths were synthesized using a strategy based on two sequential ROPs.

As a general approach, α-methoxy-ω-amino-poly(ethylene glycol) (mPEG-NH_2_) or poly(ethylene glycol) diamine (H_2_N-PEG-NH_2_) are widely used as macroinitiators for ROP of NCA monomers, resulting in the formation of block copolymers.^66,67^ For example, mPEG-NH_2_ was employed as an initiator for the synthesis of peptide conjugates by random ring-opening copolymerization (ROCP) of three NCA monomers; l-isoleucine (I), l-alanine (A), and glycine (G).^68^ Various diblock copolymers consisting of a PEG block and a random poly(l-alanine-glycine-l-isoleucine) (P(A-G-I)) block were synthesized with varying peptide chain lengths while a NCA monomer feed ratio of 1/1/1. The resulting mPEG-*b*-P(A-G-I) copolymers demonstrated the ability to respond to multiple stimuli, enabling their use in the design of hydrogels for delivery systems.

In another study, oligo(tyrosine) (Y)_*n*_-PEG conjugates (PEG-Y_*n*_) were synthesized using mPEG-NH_2_ as a macroinitiator for the ROP of O-benzyl-l-tyrosine NCA, followed by a deprotection step.^69^ The use of mPEG-NH_2_ with different molar masses and several NCA/macroinitiator ratios yielded PEG-Y_*n*_ diblock copolymers with various compositions.

**Figure 4:**
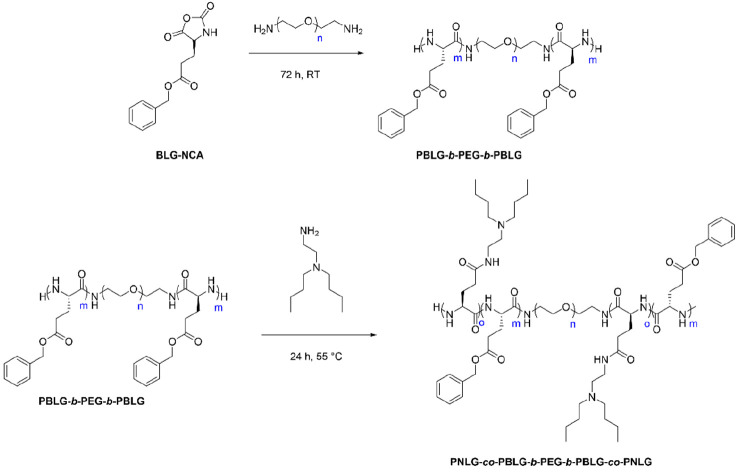
Synthesis route of the PNLG-*co*-PBLG-*b*-PEG-*b*-PBLG-*co*-PNLG polypeptide-based polymer conjugates. Adapted with permission from ref (70). Copyright 2017 Royal Society of Chemistry.

PPCs with multiple stimuli responsiveness have also been designed by the ROP of NCA monomers. Comb-polymer peptide conjugates were developed to undergo temperature- and pH-triggered gelation.^70^ The copolymers consisted of a central PEG block and two external blocks of poly[(2-(dibutylamino)ethyl-l-glutamate)-*co*-(γ-benzyl-l-glutamate)] (PNLG-*co*-PBLG). The synthesis of the peptide conjugate was performed using H_2_N-PEG-NH_2_ as the initiator for the ROP of γ-benzyl-l-glutamate-NCA (BLG-NCA) (Figure 4). After polymerization, the PBLG blocks underwent partial aminolysis to yield the ABA type PNLG-*co*-PBLG-*b*-PEG-*b*-PBLG-*co*-PNLG copolymer.

A dual-responsive hydrogel was developed by Zhu et al. using methyl-PEG-*b*-poly(O-benzyl-l-threonine) (mPEG-*b*-PBnLT) diblock copolymer.^71^ The synthesis of mPEG-*b*-PBnLT with different chain lengths was achieved by using mPEG-NH_2_ as the initiator for the ROP of NCA of the O-benzyl-l-threonine. More recently, a series of thermoresponsive diblock copolymers consisting of PEG and peptides with various block lengths and alkyl pendant groups was developed by the group.^72^ The three amino acids l-leucine, l-valine, and l-alanine, which provide isobutyl, isopropyl, and methyl pendant groups, respectively, were selected to compare the structure–property relationship of the copolymers’ self-assembly and thermoresponsiveness. A similar approach was employed for the synthesis of the diblock copolymers, using mPEG-NH_2_ as the initiator for the ROP of NCA from three amino acids.

In addition to PEG, other macroinitiators have been employed in the design of temperature-responsive polypeptide-based PPCs. For instance, bis(3-aminopropyl)-terminated poly(dimethylsiloxane) (H_2_N–PDMS–NH_2_) was employed as a macroinitiator for the ROP of BLG-NCA.^73^ By use of the H_2_N–PDMS–NH_2_ initiator with different PDMS chain lengths and varying monomer/macroinitiator ratios, ABA type PBLG-*b*-PDMS-*b*-PBLG block copolymers with multiple PDMS and PBLG block lengths were synthesized.

### 2.6 Other Designs of Peptide–Polymer Conjugates

Among the wide variety of peptides, sequences other than those mentioned above have been used in the development of temperature-responsive PPCs.^74−77^ Met-enkephalin, a peptide known for its antitumor properties, was the subject of a study in the design of thermoresponsive PPCs.^78^ The peptide was synthesized by using SPPS and modified for further polymerization. It was used as a macroinitiator to design a random copolymer based on poly((di(ethylene glycol) monomethyl ether methacrylate) and (oligo(ethylene glycol) monomethyl ether methacrylate)) through an activator generated by electron transfer (AGET) ATRP. PPCs were engineered using both natural and unnatural octa(valine) (L-V-D-V)_4_ to respond to multiple stimuli. A conjugate of poly(dimethylaminoethyl methacrylate) (PDMAEMA) and hydrophobic (L-V-D-V)_4_ exhibited responsiveness to both temperature and pH.^79^ In designing the conjugate, the peptide was chosen to provide self-assembly properties, and the polymer was chosen for its responsiveness to multiple stimuli. The PDMAEMA polymer was synthesized by atom transfer radical polymerization. A specific initiator, (2-azidoethyl)-2-bromo-2-methylpropanamide (αBIB-N_3_), was used to introduce the azide function at the polymer chain end. The (L-V-D-V)_4_-PDMAEMA conjugate was obtained by CuAAC coupling between azide-functionalized PDMAEMA and the alkyne-functionalized l,d-octapeptide on SPPS.

**Figure 5:**
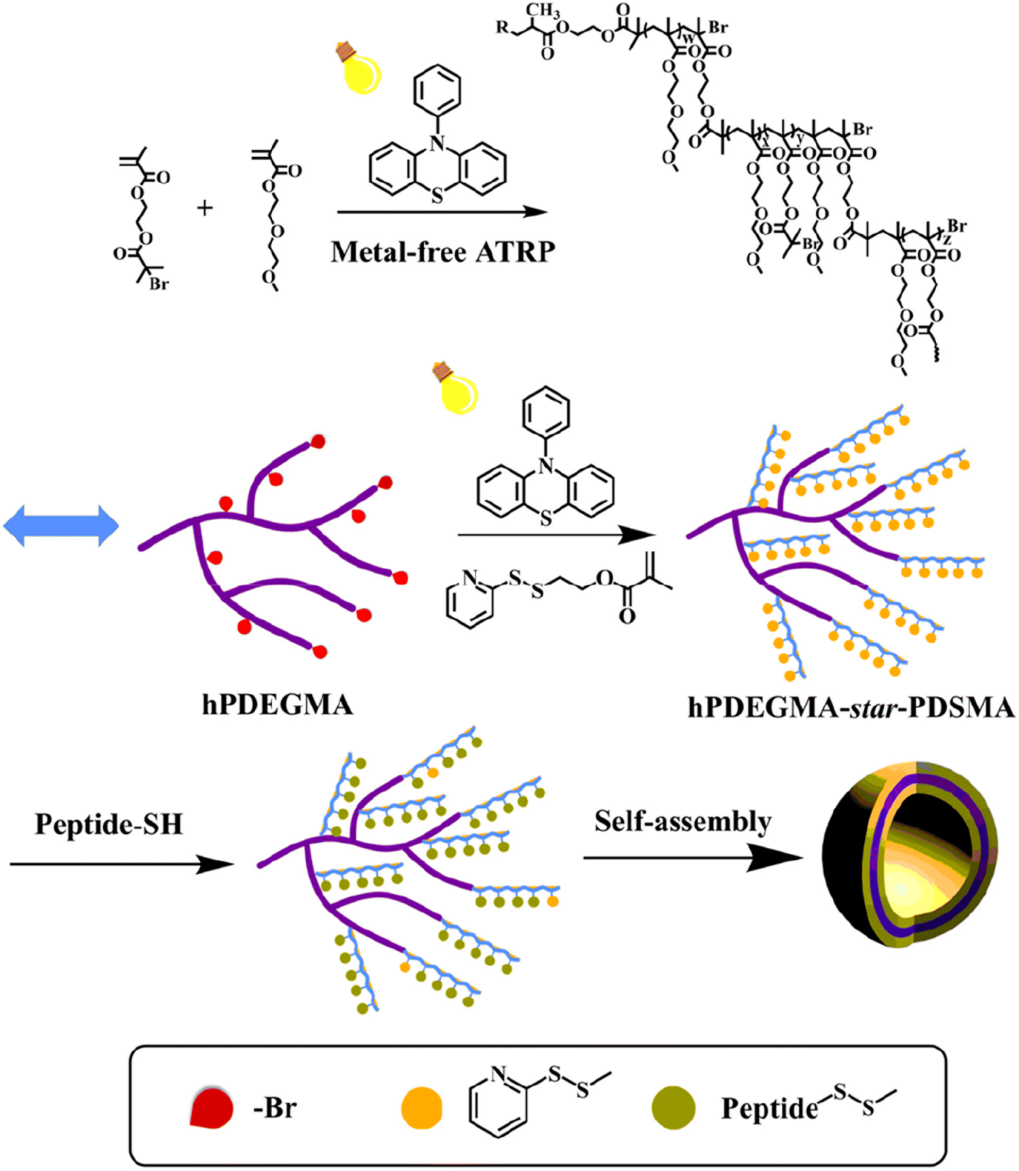
Synthetic route and self-assembly of the hyperbranched hPDEGMA-star-PGS conjugates. Reprinted with permission from ref (80). Copyright 2023 Royal Society of Chemistry.

More recently, thermoresponsive hyperbranched conjugate systems were developed to respond to temperature.^80^ Such an architecture was employed in the conjugation of glutathione (GSH) peptide with hyperbranched poly(di(ethylene glycol) methyl ether methacrylate) (hPDEGMA). As a first step, ATRP with light irradiation was employed to afford the hPDEGMA by copolymerization between a monomer and an inimer (initiator-monomer) (Figure 5). Targeting similar molar masses, three hPDEGMA with different degrees of branching (DB) were synthesized by varying the monomer/inimer ratio. A second light-mediated ATRP was performed using hPDEGMA as the initiator to polymerize pyridyl disulfide ethyl methacrylate (DSMA), yielding the hPDEGMA-*star*-PDSMA polymer. The use of the GSH peptide enabled the formation of the final hPDEGMA-*star*-PGS conjugate through an exchange reaction between the thiol of GSH and the disulfide of the PDSMA units.

Anisotropic motion in response to temperature was achieved in PPCs using two amphiphilic peptides and a thermoresponsive polymer, as reported by the Stupp group.^81^ A bromoisobutyryl group was introduced at the chain end of one of the peptides to enable ATRP. Nanorods were first obtained through the coassembly of the two amphiphilic peptides. Following a thermal annealing treatment of the nanofibers, an aqueous lyotropic liquid crystal was formed. Applying a rotational shear force to the peptide solution within a tubular mold resulted in the formation of circumferentially aligned hydrogel tubes. The modified amphiphilic peptides present in the nanofibers were used as initiators for the subsequent polymerization of oligo(ethylene glycol) and diethylene glycol methyl ether methacrylates (OEGMA and DEGMA, respectively) by ATRP. The temperature-responsive polymers were thus grafted from the scaffold surface, exhibiting an LCST transition of 35 °C. Due to the presence of a cross-linker during the ATRP, both intra- and intermolecular covalent bonds within the nanofibers were obtained. The resulting actuators were subjected to temperature changes from 25 to 70 °C in water. By variation of the temperature above or below the LCST of the polymer, several cycles of anisotropic expansion and contraction could be performed by the material.

Several studies have also explored the use of proteins to develop thermoresponsive protein–polymer conjugates.^82−85^ PEGylation of laccase with a random copolymer of poly(di(ethylene glycol) ethyl ether acrylate)-*r*-poly((ethylene glycol) methyl ether acrylate) poly(DEGEEA)-*r*-(PEGA480) provided stimuli-responsiveness to the self-assembled conjugate.^86^ In another example, hexameric tyrosine-coordinated heme protein (HTHP) was employed in the design of a temperature-responsive conjugate due to its stability toward high temperatures (denaturation at 130 °C).^87^ In the design of the conjugate, HTHP was modified with cysteine to enable the presence of a thiol function on the protein surface. The use of maleimide functionalized poly(*N*-isopropylacrylamide) (PNIPAAm) allowed for the synthesis of the HTHP-PNIPAAm conjugate.

Thermoresponsive conjugates have also been developed using oligo(ethylene glycol) (OEG) chains conjugated to peptide motifs.^88^ This approach was notably investigated in the Besenius group.^89,90^ They recently developed a *C*_3_-symmetric dendritic conjugate consisting of the triphenylalanine FFF sequence and dendritic OEG.^91^ For the conjugate synthesis, the N_3_-GFFF–OH peptide was first synthesized by SPPS. The peptide was then coupled to an amine-containing OEG dendron by an amidation reaction. The final conjugate was obtained by CuAAC coupling with 1,3,5-triethynylbenzene. This PPC was further used in temperature induced gel toughening.

In this second part, general methods for the synthesis of PPCs were presented. A summary of the general synthetic methods, such as grafting to, grafting from, and grafting through strategies, is provided. The general design features of a wide range of temperature-responsive PPCs are presented. Using these strategies, a library of various types of peptides, including ELP, CMP, amyloid-β, cyclic peptides, polypeptides, and others, can be conjugated to polymers for the preparation of temperature-responsive conjugates. The discussion highlighted the synthetic routes, characterization techniques, and thermoresponsive properties of the conjugates.

## 3 Supramolecular Assembly of Thermoresponsive Peptide–Polymer Conjugates

Supramolecular assembly describes the organization of building blocks into well-defined structures through noncovalent interactions, such as hydrogen bonding, hydrophobic, electrostatic or van der Waals interactions, π–π stacking, and metal ion coordination.^92^ Peptides are a category of molecules that have been widely studied for their self-assembly properties. Their ability to interact through intra- and intermolecular interactions allows for the formation of ordered structures, including β-sheets, helices, or coiled-coil structures.^93−95^ Various morphologies can be achieved through self-assembly, which is regulated by both thermodynamics and kinetics and can be influenced by external parameters including pH, solvent composition, or temperature.^16^ The availability of a large panel of amino acids with diverse chemical functions has facilitated the design of materials with tunable morphologies and properties. By conjugating polymers with peptide units, the diversity of structural assemblies, triggered stimuli-responsive properties, and interactive system behavior with living matter can be hugely expanded. Over the last few decades, PPCs have demonstrated versatile temperature-triggered self-assembly. The following section describes different morphologies obtained from the thermoresponsive PPCs.

### 3.1 Supramolecular Assembly of Thermoresponsive Peptide–Polymer Conjugates: Micelle, Polymersome, and Coacervate Formation

**Figure 6:**
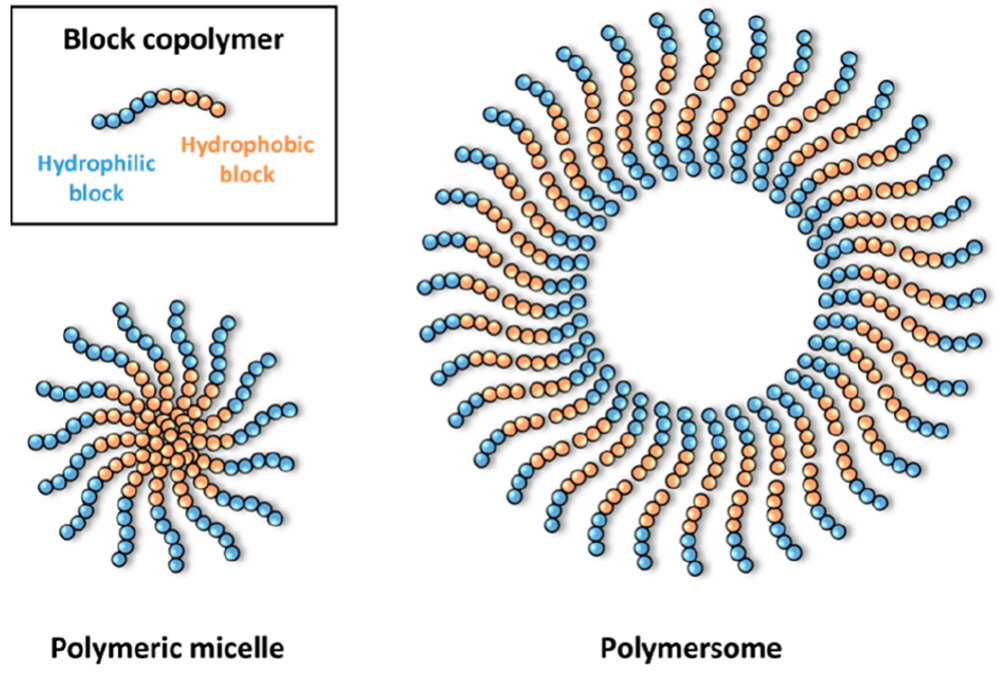
Schematic representation of a polymersome and a polymeric micelle.

The formation of micelles and polymersomes using temperature-responsive PPCs has been intensively investigated, particularly for advancements in carrier biomaterial design purposes and delivery systems. Polymeric micelles are characterized by the organized self-assembly of amphiphilic macromolecules, resulting in a core–shell structure (Figure 6). Polymersomes, on the other hand, form a bilayer-like structure enclosing a liquid inner phase. Temperature-responsive PPCs were proposed to self-assemble into these morphologies.^52^ The thermoresponsive properties and self-assembly of the amphiphilic amyloid-based PDEGA-*b*-LVFF described above were investigated in aqueous solution.^51^ Below the LCST temperature, the PPC self-assembled into micellar structures consisting of a hydrophilic polymer shell and a hydrophobic peptide core. The increase in the conjugate chain length resulted in an increase in the hydrodynamic diameter (*D*_h_) of the micelles, as expected. Moreover, by applying a temperature near the LCST of the conjugate, it was suggested that the polymeric shell dehydrated and the micelles aggregated due to loss of colloidal stability.

**Figure 7:**
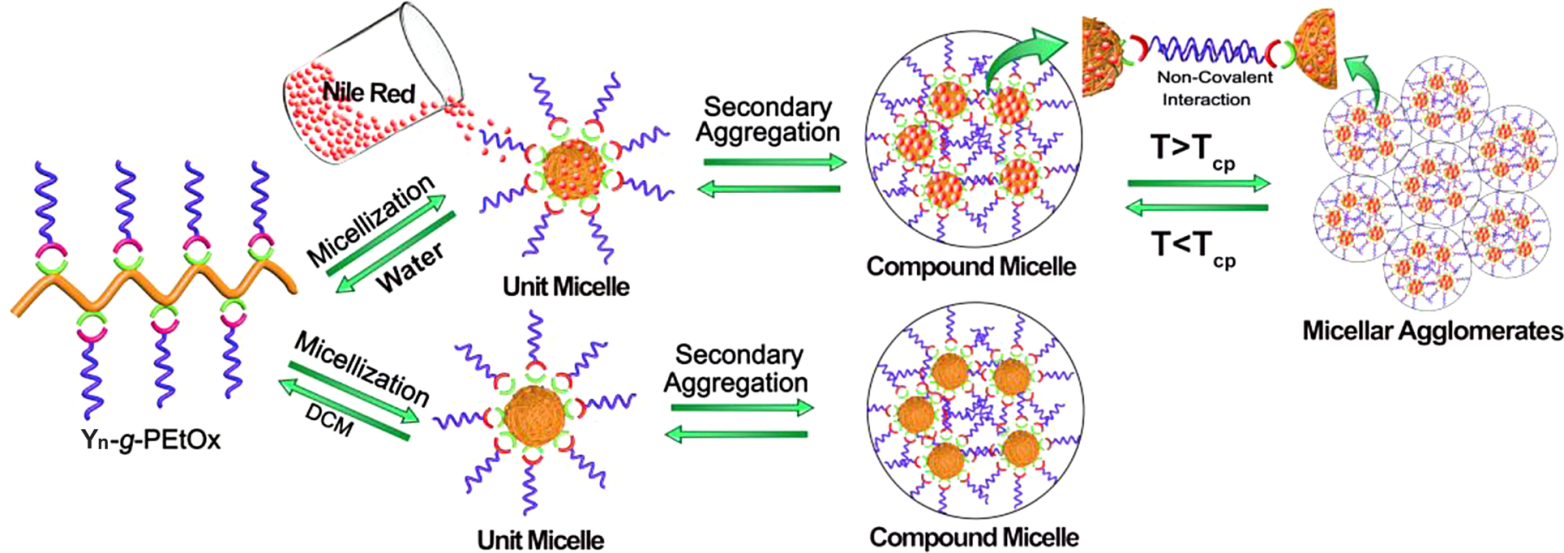
Illustration of the self-assembly behavior of the Y_*n*_-*g*-PEtOx conjugates in water and in DCM. Adapted with permission from ref (65). Copyright 2017 Elsevier.

The properties and self-assembly of amphiphilic Y_*n*_-*g*-PEtOx copolymers were evaluated in both aqueous and nonaqueous media.^65^ Y_*n*_-*g*-PEtOx exhibited solubility in water, while the solubility of the (Y)_*n*_ block was limited to high pH. A reversible LCST behavior was observed for the Y_*n*_-*g*-PEtOx copolymers in water, which was attributed to the PEtOx block. By variation of the experimental parameters, including the length of both blocks and the copolymer concentration, the cloud point (*T*_cp_) was adjusted. In organic solvent, the copolymers self-assembled into spherical micelles, which further aggregated to form compound micelles (Figure 7). Similar behavior was also observed for the copolymers in water. However, higher order and larger particles were formed, resulting from the agglomeration of the compound micelles with increasing temperature above the *T*_cp_.

The temperature-triggered self-assembly of the hexameric tyrosine-coordinated heme protein HTHP-PNIPAAm conjugate was evaluated in aqueous solution.^87^ Increasing the temperature led to the formation of spherical micelles through self-assembly of the conjugate. The PNIPAAm formed the hydrophobic core, and the HTHP constituted the hydrophilic shell of the micelle. The reversible self-assembly/disassembly of the HTHP-PNIPAAm conjugate was performed five times. In the case of laccase protein conjugated to poly(DEGEEA)-*r*-(PEGA480), nanoparticles were formed in water.^86^ Their diameter and distribution decreased with increasing temperature, resulting in more uniform nanostructures. This observation was attributed to the increase in hydrophobicity of the polymer chains.

The self-assembly of the hyperbranched hPDEGMA-*star*-PGS conjugates was studied in water.^80^ Upon increasing the temperature was increased to 37 °C, vesicles with distinct layers were formed, depending on the DB of the conjugate. In particular, hPDEGMA-*star*-PGS vesicles consisting of bilayers were formed with a lower DB, while a higher DB in the conjugate led to the formation of monolayer vesicles. The ability of PDEGMEMA to collapse at temperatures above its LCST was found to be of interest in the self-assembly of diblock conjugates. In the case of the collagen mimetic peptide-PDEGMEMA diblock conjugates, the formation of the peptide’s triple helical structure was observed.^43^ The conjugate exhibited reversible self-assembly into nanovesicles when heated to above its transition temperature (37 °C).

The influence of various experimental conditions and methods, such as solvents, stirring, or concentration, on the self-assembly of PEG-*b*-poly(ε-caprolactone) PEG-*b*-PCL block copolymer grafted with peptides containing Y–Y and RGD motifs was investigated.^96^ Sonication at 60 °C, followed by a temperature decrease enabled the formation of needles and flat micelles, triggered by the crystallization behavior of PCL. In addition, due to the hydrophobicity of the peptide and the formation of hydrogen bonds and π–π interactions, the structures containing the Y–Y-based peptide showed improved stability.

The temperature-dependent self-assembly of the E_*n*_-*b*-ELP conjugates was studied in pure water and PBS solution.^35^ With an increase in temperature, the copolypeptides showed a transition from soluble chains to macroscopic aggregates in pure water. Increasing the chain length of the (E)_*n*_ block resulted in an increase in the transition temperature (*T*_t_) and concentration dependence due to the higher hydrophilicity of the copolypeptide. In PBS buffer, a transition from soluble chains (below the *T*_t_), to aggregates (above the *T*_t_) was also observed with increasing temperature. The aggregates evolved into well-defined nanoparticles (at the critical micellization temperature CMT) and reached equilibrium. Higher temperatures (above the CMT) led to dehydration and densification of the spherical nanoparticles. Therefore, the screening of the negative charges of glutamic acid units in the shell and the compaction of the core of the micelle-like structure contribute to the thermoresponsive self-assembly of the copolypeptides in the presence of salt. The temperature-responsive behavior and self-assembly of the ELP-*b*-polysaccharide conjugates were studied in aqueous medium.^37^ By variation of the temperature, the conjugates were able to self-assemble into nanoparticles in a reversible manner. Stable and well-defined nanosized particles formed when the temperature exceeded the ELP’s *T*_t_. The influence of the structure on the thermal self-assembly properties of ELP-*b*-HA conjugates was studied across nine derivatives.^38^ Analysis of nanoparticle characteristic values, including *D*_H_, critical micellar temperature (CMT), and concentration (CMC), showed block length-dependent behavior. For instance, the hydrodynamic diameter (*D*_H_) of the nanoparticles could be increased by employing a longer ELP block. This, in turn, resulted in a decrease in the CMT value and the CMT dependence as a function of the molar concentration. Modification of the polysaccharide length also had an effect on the self-assembly properties of the conjugates. Higher *D*_H_, CMT, and CMC values were obtained by increasing the length of the HA block.

Blends of PPCs have also been used. For instance, blending of copolymers was employed in the formation of polyelectrolyte complex (PEC) micelles with temperature-responsive behavior.^97^ Such complex formation results from the self-assembly of block copolymers containing charged and neutral segments. The core of the PEC micelles was composed of the associated complementary charged segments, while the shell of the structure consisted of neutral segments. The thermoresponse in this study is provided by pNIPAAM due to its LCST behavior. The self-assembly study focused on two different systems, both consisting of the diblock copolymer poly(*N*-isopropylacrylamide)-*b*-poly(acrylic acid) (pNIPAAM–pAA) with negatively charged pAA blended with a positively charged polymer. The first system involved blending pNIPAAM–pAA with a poly(l-lysine) (PLK), while the second system involved mixing pNIPAAM–pAA with a poly(ethylene glycol)-*b*-poly(l-lysine) (PEG–PLK) conjugate. Characterization of the self-assembled structures by dynamic light scattering (DLS), transmission electron microscopy (TEM), and small-angle X-ray scattering (SAXS) revealed the formation of worm-like micelles in the pNIPAAM–pAA/PLK system. Substitution of homopolymer PLK with the PEG–PLK conjugate resulted in the formation of spherical micelles. Upon an increase in the temperature, the aggregation of both systems into larger structures is observed. However, the pNIPAAM–pAA/PEG–PLK blend maintained its self-assembled structure, while the pNIPAAM–pAA/PLK blend lost its integrity.

Temperature-responsive PPCs have also been reported to form other spherical assemblies such as coacervates. This morphology is based on liquid–liquid phase separation, resulting in the formation of a macromolecule-rich phase (coacervate) in equilibrium with the dilute phase. In a recent study, ELP-based PPCs were used in the design of thermoresponsive organelle-mimics by microfluidics.^98^ These conjugates were formed by conjugating recombinant ELP with biocompatible polymers, such as poly(ethylene glycol) (PEG) and dextran (Dex). Upon heating past their cloud point temperature (*T*_cp_), both ELP-*b*-PEG and ELP-*b*-Dex bioconjugates showed reversible self-assembly into coacervates. The study was further investigated by introducing an ELP monoblock to the ELP-based PPC. When heated to temperatures below the *T*_cp_ of the ELP-polymer conjugates yet above the *T*_cp_ of the ELP monoblock, the bioconjugates were able to self-assemble with the ELP monoblock serving as surfactants, resulting in stabilization of the coacervates.

### 3.2 Supramolecular Assembly of Thermoresponsive Peptide–Polymer Conjugates: Nanotube Formation

**Figure 8:**
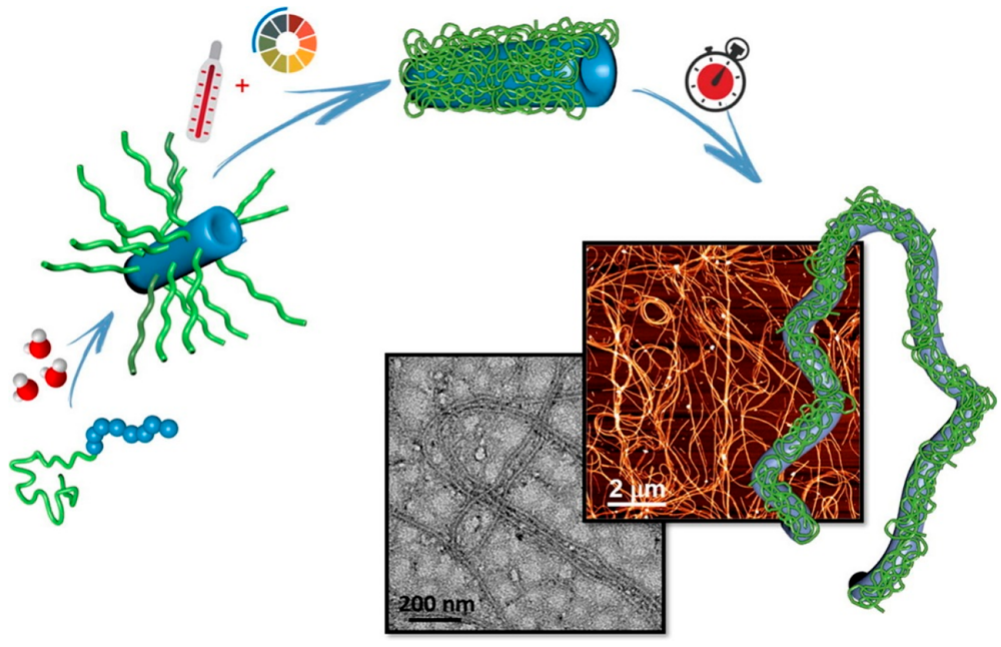
Nanotube formation based on (L-V-D-V)_4_-PDMAEMA conjugates as a function temperature and pH. Adapted with permission from ref (79). Copyright 2019 Elsevier.

The formation of channels, or nanotubes, using thermoresponsive PPCs has also been investigated. This morphology is based on a self-assembled structure with a tubular shape (hollow cylinders or channels). For instance, the multistimulus responsive properties of the (L-V-D-V)_4_-PDMAEMA conjugate on the self-assembly properties were studied in aqueous solution.^79^ Under acidic conditions, the PPC self-assembled into nanotubes consisting of a single channel with a length ranging from 0.2 to 1.5 μm at a temperature of 25 °C. It is assumed that helical peptide structures are formed through a combination of hydrophobic effects, desolvation, and van der Waals interactions, as well as hydrogen bonds between the peptides. Comparable nanostructures are observed under basic conditions at the same temperature (Figure 8). However, under these conditions, the length of the nanotubes increased to the micrometer scale with an increase in temperature beyond the LCST of the polymer. The authors suggested that the conversion of the polymer from coil to globule and desolvation between hydrophobic polymer chains and the isopropyl pendant functionalities of the peptide occurred at high temperatures, thereby modifying the conformation of the peptide. By lowering the temperature below the LCST of PDMAEMA, the nanotubes showed good stability and maintained their length. Shorter nanotubes, with a length between 0.2 and 0.5 μm, were formed by applying a mechanical force via sonication. Acidification of the medium also contributed to decreasing the length of the nanotubes.

The study of the self-assembly properties of the (pEtOx_40_)_2_–CP conjugates in water showed the formation of nanotubes.^58^ Upon increasing the temperature to the conjugate cloud point temperature, these nanostructures shifted to microparticles. Lowering the temperature allowed for reversible conformational transitions. Furthermore, the ability of the cyclic peptide conjugates to form nanotubes in lipid bilayers was investigated.^59^ Indeed, the self-assembly of the cyclic peptide coupled with hydrophilic or hydrophobic polymers takes place as channels in phospholipid bilayers. The authors observed a dependency on channel formation with the polymer structure conjugated to the peptide. The temperature-responsive behavior of the conjugate was also investigated by using PNIPAAm as the polymer component. The LCST behavior of PNIPAAm allowed the conjugate to regulate the channel constitution as a function of temperature, highlighting its potential as a thermoresponsive transbilayer channel.

### 3.3 Supramolecular Assembly of Thermoresponsive Peptide–Polymer Conjugates: Helical Structures and High-Order Assemblies

Temperature-responsive PPCs have also been shown to self-assemble into higher-order helices such as triple helices or superhelices. Zhu et al. reported on the formation of superhelical structures composed of PPCs.^99^ By blending a PBLG homopolymer with a PBLG-*b*-PEG diblock copolymer in aqueous solution, helices composed of numerous strands were formed. The influence of various experimental parameters, including the PBLG-*b*-PEG weight fraction or polymer concentration, on the strand number of the self-assembled structure was evaluated. Increasing the temperature notably allowed an increase in the number of strands in the structure due to a decrease in the helical angles induced by the arrangement of the phenyl groups of the PBLG side chains. Furthermore, the authors proposed a mechanism for the self-assembly of the polymers into superhelical structures.

**Figure 9:**
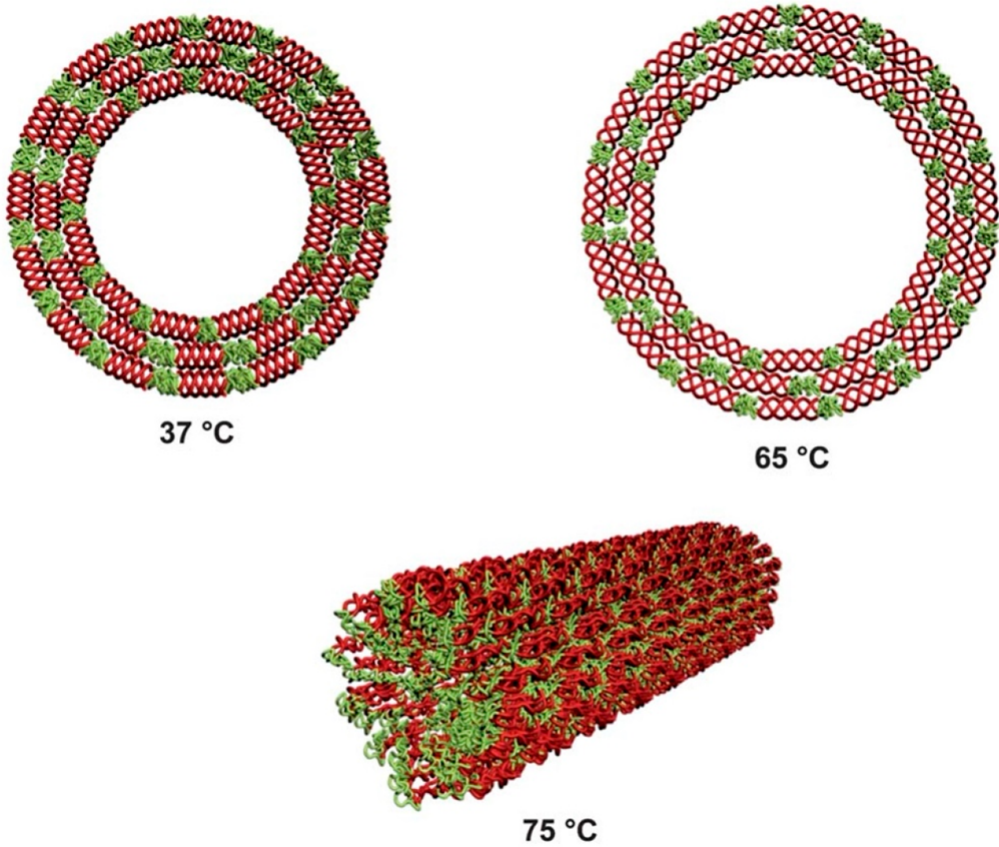
Self-assembled superstructures of the PDEGMEMA–collagen mimetic peptide–PDEGMEMA conjugate at different temperatures. Reprinted with permission from ref (42). Copyright 2012 Royal Society of Chemistry.

Multiple self-assemblies of thermoresponsive PPCs can also be obtained by manipulating the experimental parameters. The self-assembly properties of collagen-based conjugates were investigated as a function of the temperature. For instance, the PDEGMEMA–collagen mimetic peptide–PDEGMEMA triblock copolymers exhibited dual responsiveness and diverse supramolecular structures.^42^ Analysis by circular dichroism (CD) first revealed the self-assembly of the peptide into a triple helix. The elevation of the temperature above the conjugate LCST (≈ 35 °C) resulted in polymer collapse and enhanced thermal stability of the collagen mimetic block. Investigation of the morphological structure of the conjugate above the LCST revealed the formation of spherical aggregates with a diameter in the micrometer range (Figure 9). Peptide unfolding at a higher temperature (65 °C) resulted in an increase in the diameter of the structure. A further increase in temperature to 75 °C led to the conversion of the spheres into fibrils.

In summary, this section presents the temperature-triggered supramolecular assembly of PPC systems into diverse structures. The wide range of thermoresponsive PPCs allowed the formation of nano- and micrometer-sized morphologies including spherical micelles, vesicles, coacervates, channels, or tubes. We have highlighted thermodynamic driving forces and parameters as well as kinetic effects while focusing on the temperature-dependent self-assembly phenomena between the peptide and polymer segments in the PPCs. In some cases, these are driven by cooperative effects but can also be of opposing character, leading to frustrated systems.

## 4 Thermogelation of Thermoresponsive Peptide–Polymer Conjugates

Supramolecular gels are physically cross-linked networks obtained from molecular or macromolecular aggregation.^100−104^ Physical gels involve noncovalent interactions, including hydrogen bonding, π–π stacking, ionic, or van der Waals forces, supported via desolvation and the hydrophobic effect. In contrast to chemical covalent networks, supramolecular gels exhibit lower chemical and mechanical stabilities, which can limit their range of applications. On the other hand, the main advantage of supramolecular networks lies in their reversible assembly/disassembly, stimuli-responsive properties, and opportunities for the design of interactive material design features. Various supramolecular gels have been reported that make use of external stimuli such as pH, light, oxidative or reductive stress, and temperature. Thermogels are of great interest, particularly in the biomedical field, thanks to their ability to undergo a sol–gel transition upon an increase or decrease in temperature, an external trigger that is generally more biocompatible than chemical cross-linking methods if operated in an appropriate temperature window. Due to the availability of a wide range of amino acids and polymers, the temperature window for thermogelation can be finely tuned. Key factors include the ability of peptides to form secondary structures (such as β-sheets and α-helices) as well as the thermoresponsive behavior of some peptides and polymers. The LCST behavior of polymers or peptides can be notably influenced by changes in the amphiphilic balance. In general, an increase in hydrophobicity in the peptide or polymer decreases the LCST, while an increase in hydrophilic groups increases the LCST.^105^ Thermoresponsive PPCs have shown particular relevance in the formation of hydrogels thanks to their broad versatility, multifunctionality, and ability to tune the biologically relevant window for on-set temperatures.^89^ This section highlights the driving forces embedded in the polymer- and peptide-blocks or segments of different PPCs and their role in the thermogelation process.

### 4.1 Thermogelation of PPCs Based on Collagen-Mimetic and Elastin-like Peptides

The triple helix formation of collagen-based polymer conjugates enabled their use as cross-linking motifs in the formation of hydrogel networks. Three collagen-PEG conjugates were designed using collagen sequences proline–hydroxyproline–glycine (POG) with 7 to 9 POG repeating units coupled to an eight-arm star PEG polymer.^106^ At room temperature, the assembly of the triple helix resulted in the formation of a physically cross-linked hydrogel. The thermal properties of the hydrogel could be tuned depending on the POG length used in the conjugate. Indeed, the stability of the hydrogel toward temperature was improved by increasing the peptide chain length and, thus, the stability of the triple helix assembly.

The gelation process of various RHC-chitosan hydrogels was also studied and compared to that of chitosan hydrogels.^44^ The gelation temperatures of the hydrogels were determined by rheological measurements in a temperature range from 10 to 50 °C. Samples, including chitosan hydrogel and recombinant collagen-peptide(RHC)-chitosan hydrogels with several chitosan/RHC ratios (2/1, 1/1, and 1/2), were examined. Conjugation of RHC on chitosan resulted in reduced gelation temperatures from 27 °C for the chitosan hydrogel to a temperature range from 24 to 15 °C for the RHC-chitosan hydrogels. The authors hypothesized that the conjugation of RHC, in comparison to that of chitosan hydrogels mainly promoted hydrogen bonding and hydrophobic interactions during the gelation process. Enhanced mechanical strength, stability, and water vapor transmission rate (WVTR) were also achieved with RHC-chitosan hydrogels compared to a chitosan hydrogel.

**Figure 10:**
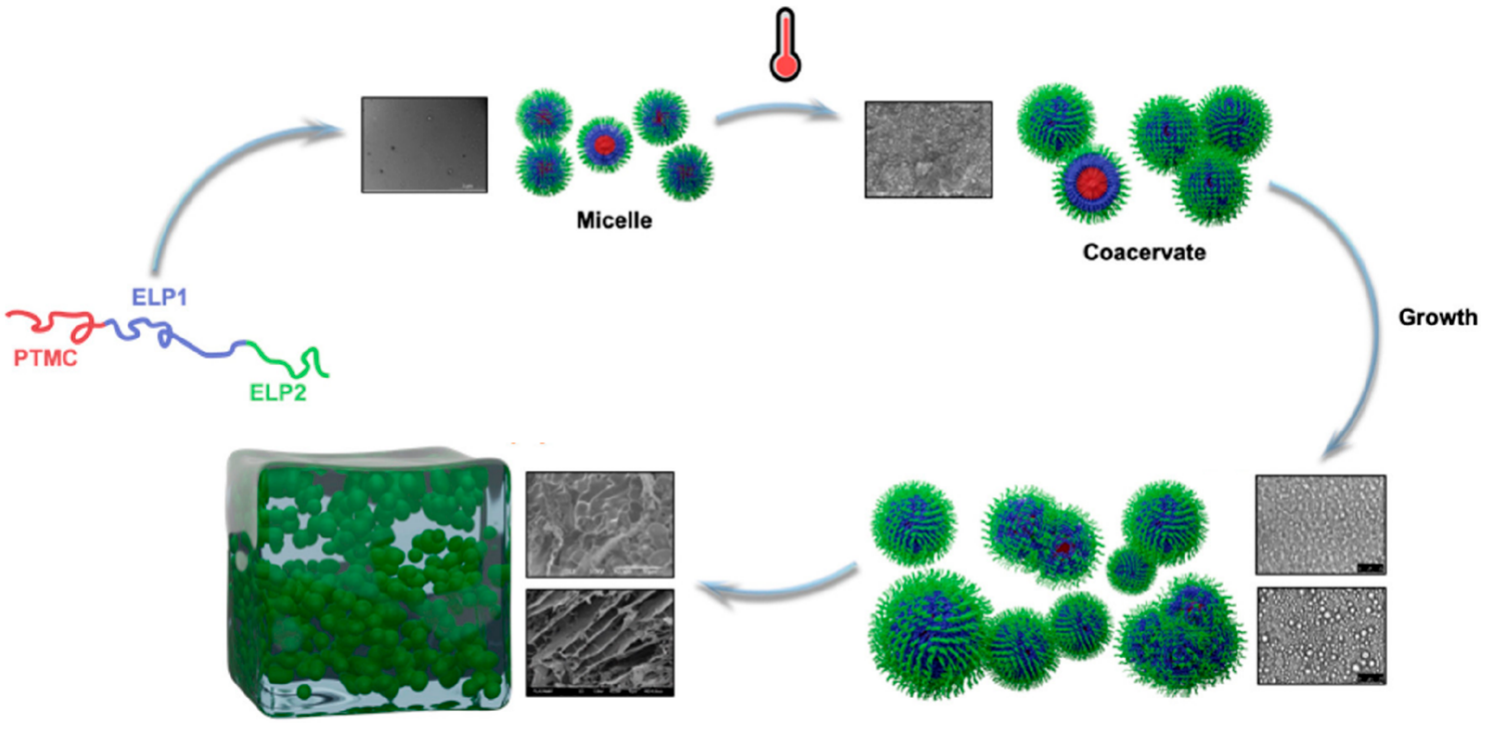
Hydrogel formation based on ELP containing ABC terblock copolymers. Adapted with permission from ref (36). Copyright 2021 American Chemical Society.

In the category of biomimic peptides, ELPs were also used to develop hydrogels due to their temperature-responsive behavior.^34^ The thermal self-assembly behavior and gelation process of the ABC terblock copolymer PTMC_30_-*b*-MW-(ELP[V_3_M_1_-60])-(ELP[I_1_-20])-C(N-EtSucc) were evaluated in ultrapure water.^36^ Micelles with a diameter of 10 to 60 nm were observed at low temperatures (below the T_t_) and dilute conditions (0.1–0.3% w/v). Upon increasing the temperature above the T_t_ value, coacervates composed of larger particles with diameters of 200–300 nm were subsequently formed. At a higher terblock copolymer concentration (4% w/v), the system led to the formation of a micrometer-sized particle network, resulting in a free-standing and thermoreversible hydrogel (Figure 10). The authors suggested the potential role of PTMC as physical cross-links, contributing to the stabilization of the network

### 4.2 Thermogelation of β-Sheet and α-Helical Encoded PPCs

The thermoresponsive properties and biocompatibility conferred by PEG have promoted its use in the development of hydrogels. Several thermogels composed of peptide-PEG based conjugates have been investigated.^67,107,108^ The amphiphilic PEG2000-Y_6_ conjugate exhibited thermogelation behavior and sol-to-gel transitions in water between 25 and 50 °C, keeping the conjugate concentrations between 0.25 and 3.0 wt %.^69^ Various parameters, such as the hydrogen bonds of the phenolic groups of the oligo(tyrosine) and β-sheet structures as well as the amphiphilic balance of the conjugate, played a key role in the gelation process of the conjugate. Investigation of the hydrogel constitution using cryogenic transmission electron microscopy (cryo-TEM) showed the formation of a fiber network. Using circular dichroism spectroscopy, the onset thermogelation process was investigated, suggesting a delicate hydrophobic/hydrophilic balance, a temperature-induced dehydration of PEG segments, and an increased packing of β-sheet domains to be at the origin of the sol-to-gel transition.

**Figure 11:**
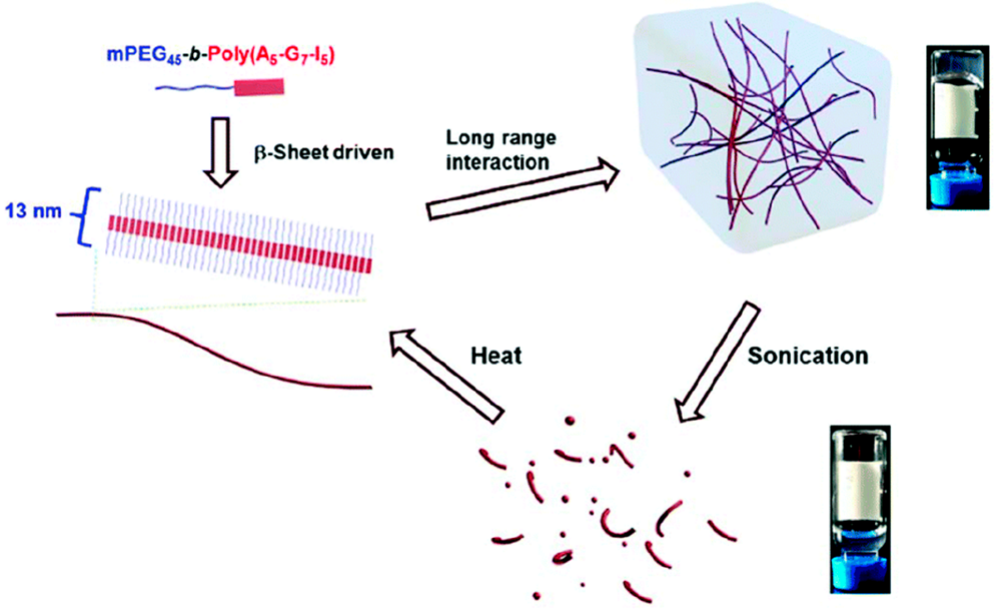
Illustration of the mechanical and temperature-responsive hydrogel based on the diblock mPEG-*b*-poly(A-G-I) copolymer. Reprinted with permission from ref (68). Copyright 2017 Royal Society of Chemistry.

The study of the self-assembly and gelation process of the diblock mPEG-*b*-poly(A-G-I) copolymer was found to be dependent on the application of mechanical, thermal, or enzymatic stimuli.^68^ Structural analysis of the copolymer in an aqueous solution revealed a predominance of β-sheet structures, resulting from the peptide block. Upon increasing temperature, the β-sheets assembled into nanofibrils through supramolecular interactions, leading to the formation of a hydrogel (Figure 11). The resulting gel could be converted back into a solution by the application of a mechanical stimulus (sonication), leading to destruction of the hydrogel networks. According to transmission electron microscopy (TEM) analysis, the initially well-defined nanofibrils were converted to shorter nanofibrils and spherical aggregates following sonication. The reversible stimuli-responsive sol–gel transitions of the deblock copolymers also appeared to be repeatable. A critical gelation temperature range (*T*_gel_) was determined by adjusting both the random polypeptide block length and the copolymer conjugate concentration. The utilization of specific enzymes also demonstrated the ability to degrade the hydrogel by targeting the peptide segments. Furthermore, the stimuli-responsive properties of the diblock copolymer showed their capability to enclose and deliver the therapeutic drug naproxen. Note that other thermoresponsive gels have been reported using peptide-PEG and peptide-PNIPAAm conjugates, which are formed through fibril structures.

The gelation behavior of mPEG-*b*-PBnLT diblock copolymers in aqueous solution was also investigated as a function of temperature by Zhu et al.^71^ Dual-responsiveness was achieved with a reversible gel-to-sol-to-gel transition upon increasing the temperature. By variation of the experimental conditions, such as the PBnLT block length or copolymer concentration, the transition temperatures could be adjusted. Moreover, investigation of the self-assembly revealed the transition of nanofibrils to spherical aggregates, leading to a gel-to-sol transition at lower temperatures. Such a transition resulted from the disassembly of the β-sheet structures. However, at even higher temperatures, PEG dehydration dominates and induces the sol–gel transition. The group also evaluated the self-assembly and thermogelation of diblock PEG-peptide copolymers in water as a function of the hydrophobicity, size, and flexibility of the peptide side groups.^72^ The methyl group in the amino acid l-alanine stands out as the smallest in size, with the lowest hydrophobicity and flexibility among the three substituents studied (alanine, valine, and leucine). The diblock copolymer consisting of alanine exhibited predominantly random coil structures. Spherical nanostructures with filaments were formed as a result of the aggregation of random coils. Increasing the length of the alanine chain, and consequently, its hydrophobicity, resulted mainly in the formation of the β-sheets, which further led to the formation of nanofibers. Similar behavior was observed for the copolymers containing valine units, leading to increased hydrophobicity, attributed to the isopropyl group. In contrast, the isobutyl group of leucine displays the greatest steric demand and the highest level of flexibility. Although this substituent possessed the highest hydrophobicity, the hindrance in the β-sheet stacking seemed to induce an irregular morphology and precipitation for leucine-based copolymers. The thermogelation of the copolymers was also evaluated in relation to the different substituents. Upon increasing the temperature, PEG dehydration influenced the gelation of copolymers comprising valine units. In the case of copolymers with alanine units, both PEG dehydration and the transition from random coils to β-sheets were involved in the gelation process.

**Figure 12:**
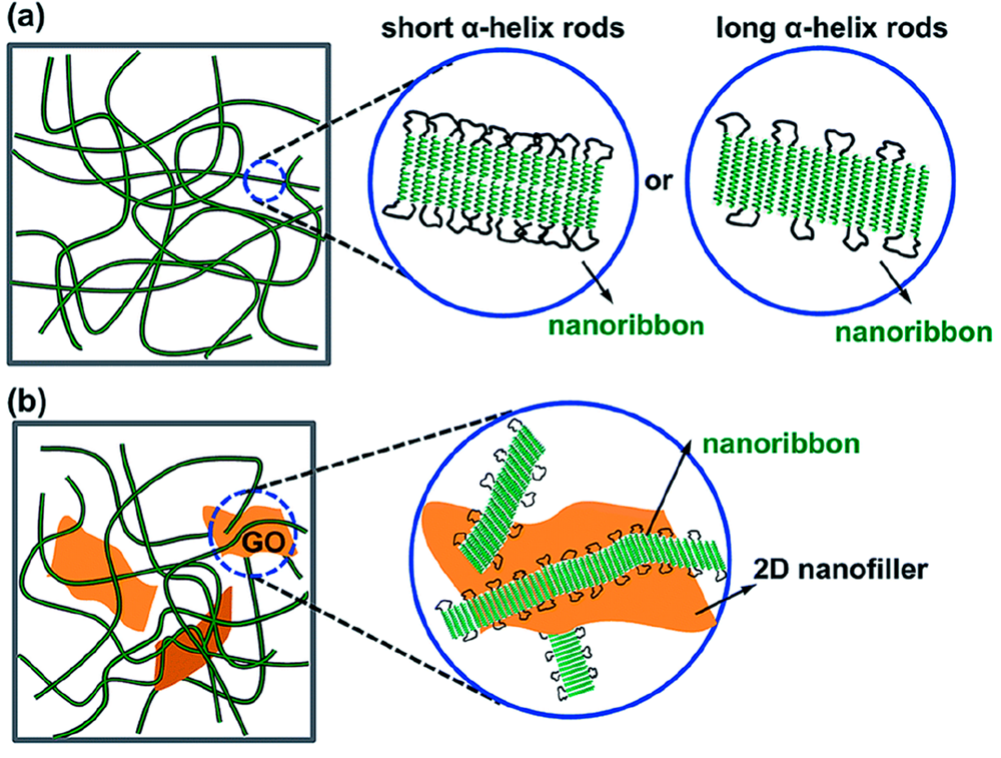
Schematic representations of the PBLG-*b*-PDMS-*b*-PBLG (A) and GO-*g*-PBLG/PBLG-*b*-PDMS-*b*-PBLG fibrillar gel networks (B). Reprinted with permission under a Creative Commons CC BY 3.0 DEED from ref (73). Copyright 2017 Royal Society of Chemistry.

The response of PNLG-*co*-PBLG-*b*-PEG-*b*-PBLG-*co*-PNLG copolymers to stimuli was analyzed with respect to temperature and pH.^70^ At room temperature (25 °C) and pH ≤ 6.4, a copolymer solution with low viscosity was observed. By increasing the temperature was increased to 37 °C and the pH was adjusted to 7.4, the copolymer exhibited a sol–gel transition. The variation in PNLG-*co*-PBLG block composition allowed modulation of the gel properties. The formation of temperature-responsive gels was also investigated for other conjugates consisting of PBLG. Thermogelation tests were conducted and assessed on PBLG-*b*-PDMS-*b*-PBLG triblock copolymers of different chain lengths to determine their properties.^73^ In toluene, the ABA type copolymers formed a solution at a high temperature of 90 °C, transitioning to a gel state as the temperature decreased. The sol–gel transitions of the copolymers exhibited thermoreversibility. The gelation parameters could be varied depending on the length of the peptide block. An increase in *T*_gel_ and a decrease in the critical gelation concentration (*C*_gel_) of the ABA copolymer were observed as the length of the helices of the peptide from the PBLG block increased in length. The gelation process was induced by the copolymer assembly, resulting from the stacking of the peptide helices and π–π interaction. Analysis of the PBLG-*b*-PDMS-*b*-PBLG gel structures showed the formation of nanoribbons (Figure 12A). In addition, graphene oxide (GO) sheets were functionalized onto the peptide and the resulting GO-*g*-PBLG was mixed with the triblock copolymer for gel formation (Figure 12B). The resulting hybrid organogel maintained its reversible thermogel properties while reducing the *C*_gel_ and enhancing its mechanical properties.

Recently, injectable hydrogels were designed using thermoresponsive PPCs based on a VFFA peptide inspired by the amyloid Aβ(1–42) sequence.^109^ The peptide self-assembled into nanofibers, which could be switched to twisted bundles by increasing temperatures. These nanostructures were cross-linked with an aldehyde-functionalized thermoresponsive polymer consisting of poly(NIPAM-*co*-formyl phenyl acrylate) using Schiff base chemistry. Upon increasing the temperature was increased to 37 °C above the LCST of the conjugates, sol–gel transitions occurred, allowing for the formation of hydrogels with good injectability, biocompatibility, and cellular proliferation properties.

### 4.3 Thermogelation of Ionic PPCs

PNIPAAm is a temperature-responsive polymer that has been extensively investigated for its ability to form a gel in response to temperature changes. The octapeptide repeat of phenyl alanine (F), glutamic acid (E), and lysine (K) FEFEFKFK has been reported to form a reversible thermogel through a 3D β-sheet fibrillar network. Stoica et al. reported the dual thermosensitivity of PNIPAAm-FEFEFKFK conjugates with very high PNIPAAm content of ≈84 wt %.^110^ At room temperature, the conjugate formed a self-supporting, semitransparent gel using peptide–polymer conjugate concentrations of 200 g/L. The gel became turbid at ≈30 °C due to the LCST behavior of the PNIPAAm polymer block. By adjusting the maximum heating temperature, the gel was able to return to a semitransparent gel or solution state upon cooling. The hydrogel properties were further assessed by blending the PNIPAAm-FEFEFKFK conjugate with the FEFEFKFK peptide or doping with the conjugate.^111,112^

In summary, we have presented and discussed numerous PPCs exhibiting thermogelation processes with reversible network formation properties. Physical gels, or hydrogels, were formed by various noncovalent interactions such as hydrogen bonding, π–π stacking, Coulomb interactions, van der Waals interactions, desolvation, and hydrophobic effects. In this section, we highlight the temperature-triggered formation or disassembly of various PPC hydrogel networks. Similar to the previous section, where intermolecular interactions drive the formation of nanostructured morphologies, the enthalpic and entropic driving forces embedded in the peptide and polymer domains can work in cooperation to reinforce each other, or have opposing effects, leading to intriguing systems involving multiple gel-to-sol-to-gel transitions. These properties further highlight the unique advantages of combining biomimetic with fully synthetic polymers for the design of multifunctional building blocks and applications as cellular scaffold materials or injectable platforms.

## 5 Applications

The ability to form well-defined architectures, combined with tunable functionalities and properties, enables the use of PPCs in a wide range of applications.^2,11,12^ PPCs have demonstrated their potential in various biomedical applications, including tissue engineering, drug delivery systems, and biosensing, among others, as well as in nano- and biotechnologies. By controlling the properties of responsive conjugates on demand, the applicability of responsive PPCs in biomedical areas has been diversified. In this last section, we discuss a few selected examples that illustrate past achievements and ongoing developments of thermoresponsive PPCs and aim to show their potential in biomedical fields for *in vivo* and *in vitro* applications.

A number of delivery systems based on thermosensitive PPCs have been developed in recent years. For instance, a stimuli-responsive gel was designed to release the anticancer drug paclitaxel by Garripelli et al.^113^ Conjugates were designed using G–P–V–G–L–I–G–L, a peptide that is degraded by matrix metalloproteinases (MMPs) overexpressed in tumor tissue. The diamine functionalized octapeptide was conjugated to standard Pluronic copolymers using a chain extension strategy to yield multiblock copolymers and to integrate the well-known thermoresponsive properties of polyethers. The resulting conjugates were able to form reversible thermogels in aqueous media. The gelation transition temperatures of the conjugates were modulated as a function of concentration and could notably occur at body temperature. In addition, MMP allowed for degradation and in *vitro drug* release from the gel.

To demonstrate the potential of the copolymer PNLG-*co*-PBLG-*b*-PEG-*b*-PBLG-*co*-PNLG as a delivery system for human growth hormone (hGH), *in vitro* and *in vivo* tests were conducted on Sprague–Dawley (SD) rats.^70^ The *in vitro* studies showed good biocompatibility of the peptide conjugate without cytotoxicity. Moreover, copolymer solutions could be injected subcutaneously into the back of the rats and immediately turned into gels. Six weeks after the injection, complete biodegradation of the biocompatible gels occurred. Following these results, anionic hGH was mixed with the cationic copolymer, resulting in the formation of a complex through ionic and hydrophobic interactions provided by the PNLG-*co*-PBLG blocks. The gel composed of the copolymer and hGH was also tested *in vivo* and showed promising results as a delivery system by reducing burst release and sustaining hormone delivery over a week.

Polymer conjugates have recently been used in the development of pH- and temperature-responsive hydrogels for the sustained release of urate oxidase (Uox).^114^ This therapeutic protein is of particular interest for the treatment of hyperuricemia, which can lead to the development of diseases such as arthritis or gout. Responsive hydrogels were developed to ensure protein delivery based on interactions between the protein and peptide-containing hydrogels. The Uox protein was conjugated to human serum albumin (Uox-HSA), which can specifically interact with albumin-binding peptide (ABP). The ABP was then conjugated to a pH- and temperature-responsive poly(ethylene glycol)-poly(β-amino ester urethane) copolymer, which includes basic (hydroxyethyl)piperazine in the monomer feed, finally affording the PEG-PAEU-ABP conjugate that was used to form the hydrogel. As demonstrated by *in vivo* and *in vitro* studies, injection of the hydrogel solution results in gel formation due to an increase in pH and temperature. The hydrogel also demonstrated biocompatibility and degradability. Furthermore, the introduction of Uox-HSA into the PEG-PAEU-ABP-based hydrogels allowed a prolonged release of Uox and therapeutic benefits.

The use of temperature-responsive PPCs for wound treatment has also been particularly investigated. The arginine-glycine-aspartic acid (RGD) peptide is a widely studied molecule known for its ability to bind to cell integrin receptors and adhesion properties^115^ that has been conjugated to temperature-responsive PPCs. Polyisocyanopeptide-based (PIC) hydrogels were developed for potential application in wound healing, as demonstrated in an *in vivo* study.^116^ The PIC polymer exhibits a helical structure with pendant OEG chains and forms a reversible gel as a function of the temperature. The PIC polymer was further functionalized with the RGD peptide motifs through “click” chemistry. Both the PIC and the PIC conjugate showed almost identical sol–gel transitions at temperatures above 15–16 °C. When the polymers were applied to a mouse wound, both materials exhibited similar properties, with respect to the closure rate, the collagen expression, or the granulocyte decrease. These results demonstrate the potential of functionalized PIC hydrogels as biomaterials for wound treatment applications. Temperature-responsive PICs have further been used as hydrogel networks in organotypic 3D cell culture.^117^

**Figure 13:**
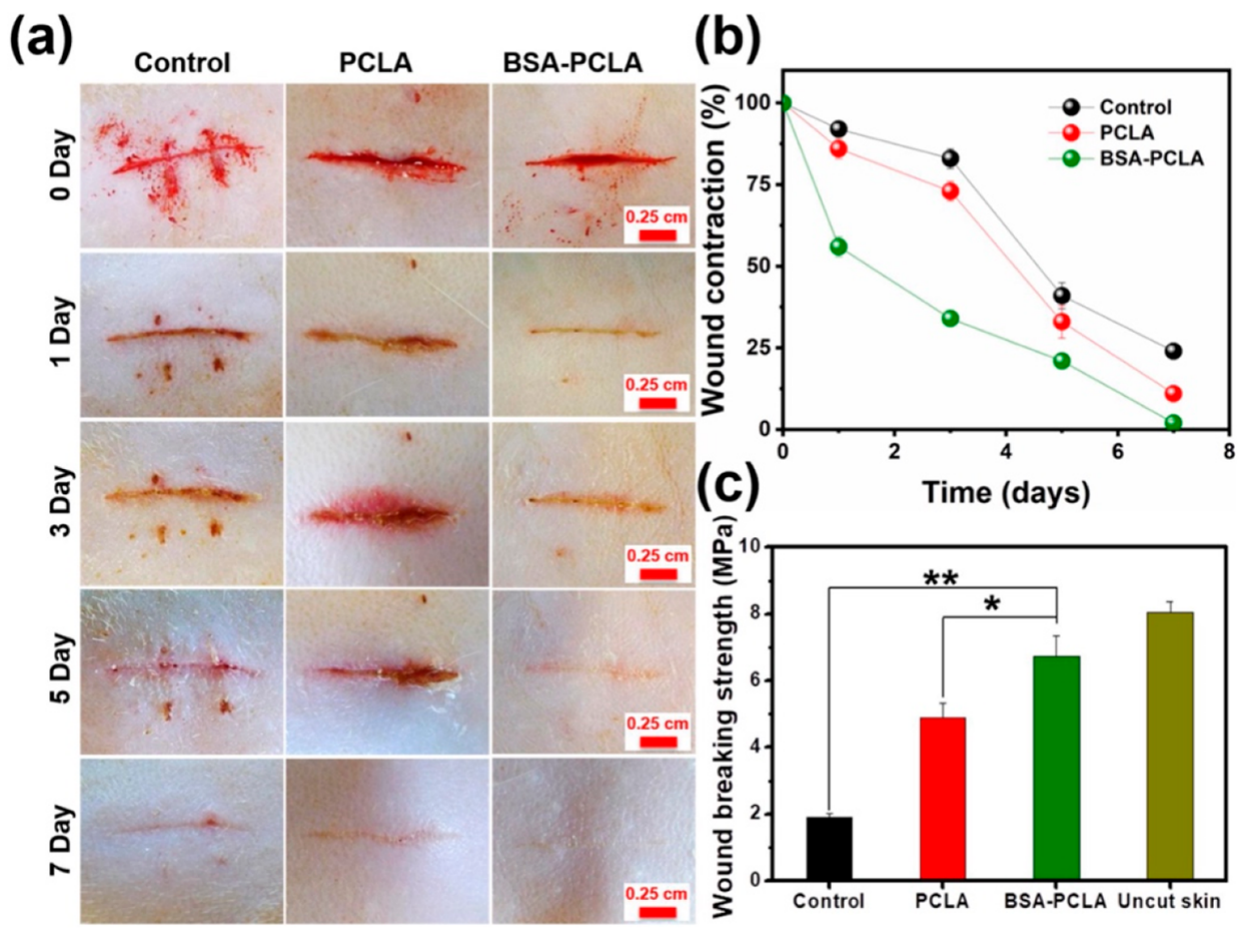
Wound treatment with the different hydrogels. (A) Photographs of the wounds. (B) Wound contraction over time with the different hydrogels. (C) Wound breaking strength for the hydrogels and uncut skin. Reprinted with permission from ref (118). Copyright 2021 Elsevier.

In another study, bovine serum albumin (BSA) protein was incorporated into a triblock copolymer to develop an injectable and temperature-responsive hydrogel for wound healing.^118^ The BSA protein was conjugated to the thermoresponsive triblock copolymer poly(ε-caprolactone-*co*-lactide)-*b*-poly(ethylene glycol)-*b*-poly(ε-caprolactone-*co*-lactide (PCLA) by the Michael addition reaction, resulting in the BSA-PCLA conjugate. At 37 °C, a hydrogel composed of the BSA-PCLA conjugate was formed owing to the increase in temperature. The temperature-responsive conjugate showed good injectability and *in vivo* gel formation in SD rat models as well as good stability, biocompatibility, and tissue adhesiveness. In addition, the BSA-PCLA hydrogels were almost completely degraded after 5 weeks. Application of the bioconjugate hydrogel to an SD rat wound improved the healing properties compared to the BSA-free hydrogels. The wound healing treatment is illustrated in Figure 13.

Recently, *in vitro* and *in vivo* studies of RHC-polysaccharide hydrogels were conducted to highlight their potential as a treatment for burn wounds.^44^ The studies indicated improved cellular bioactivity with higher RHC conjugation. When applied to burns in an SD rat model, the biocompatible hydrogels were found to promote wound healing by enhancing vessel formation, as well as cell infiltration.

**Figure 14:**
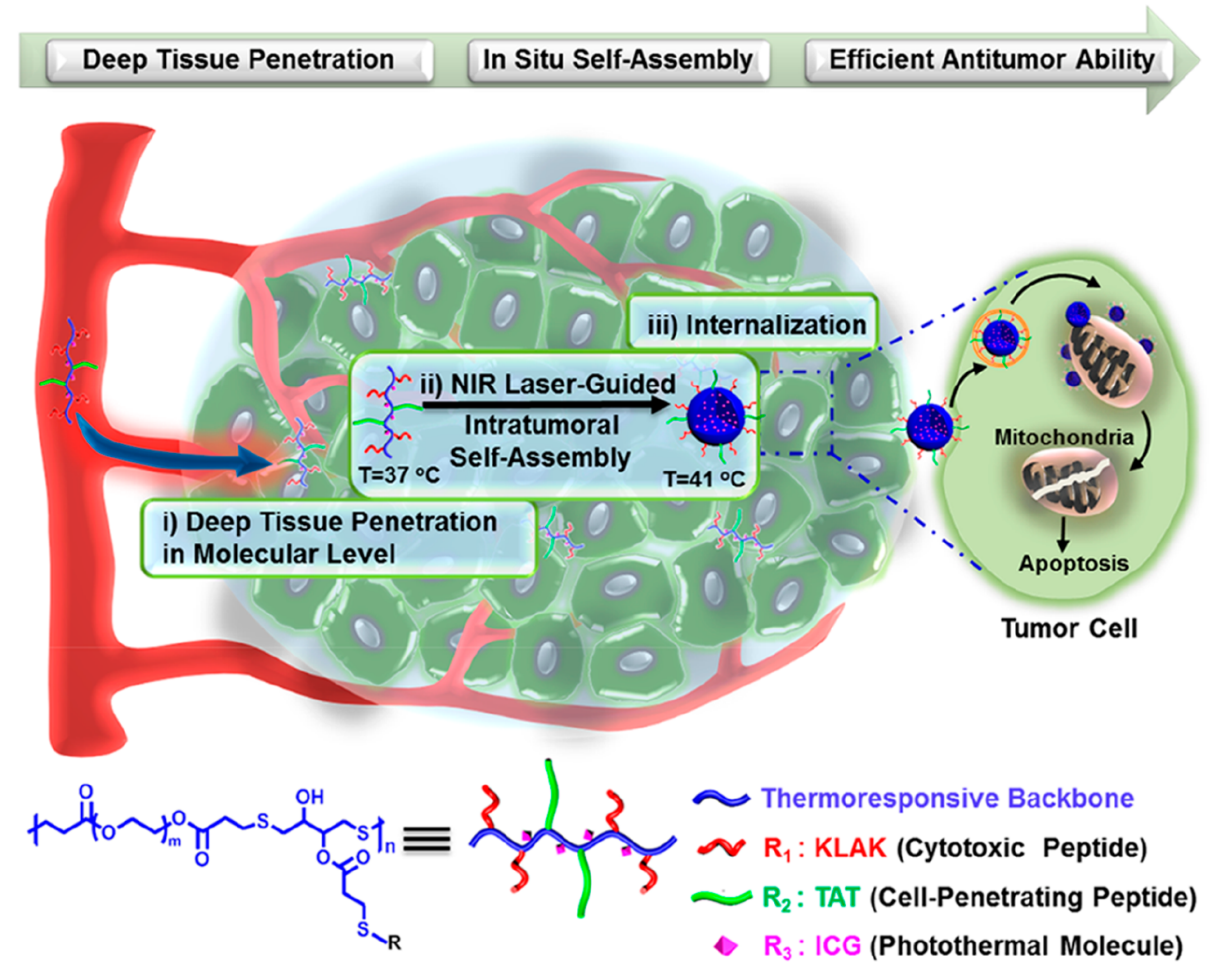
Schematic representation of the tumor treatment strategy using functionalized PPCs and NIR laser irradiation. Reprinted with permission from ref (121). Copyright 2018 American Chemical Society.

Temperature-responsive PPCs have also been evaluated for their potential applications in tumor therapy. PNIPAAm and functional paring motif conjugates, integrating a human epidermal growth factor receptor-2 (HER2) targeting peptide (CGKGGMSRTMSG), were reported as potential temperature-responsive materials in tumor therapy to prevent cancer cell proliferation.^119^ Investigations were also led on monitoring in tumor therapy.^120^ Peptides with responsive and penetrating motifs, as well as a signal molecule, were conjugated to the thermosensitive PNIPAAm. Due to their phase transition, the conjugates can form nanoaggregates in cells. A series of conjugates were synthesized, enabling improved retention effects and demonstrating advantages in tumor therapy monitoring. Another study investigated the treatment of tumors by developing polymer–peptide conjugates that improve tissue penetration and self-assembly triggered by the use of a near-infrared (NIR) laser.^121^ For this purpose, thermoresponsive poly(β-thioester) was conjugated with an NIR photothermal molecule and peptides that provide cell penetration and cytotoxicity. The conjugate allowed penetration into the tumor, followed by self-assembly into nanoparticles through NIR irradiation (Figure 14). The temperature increase induced by NIR irradiation led to the polymer collapsing and forming nanoparticles. The accumulation of nanoparticles in the tumor and their penetration into cells resulted in cell apoptosis

**Figure 15:**
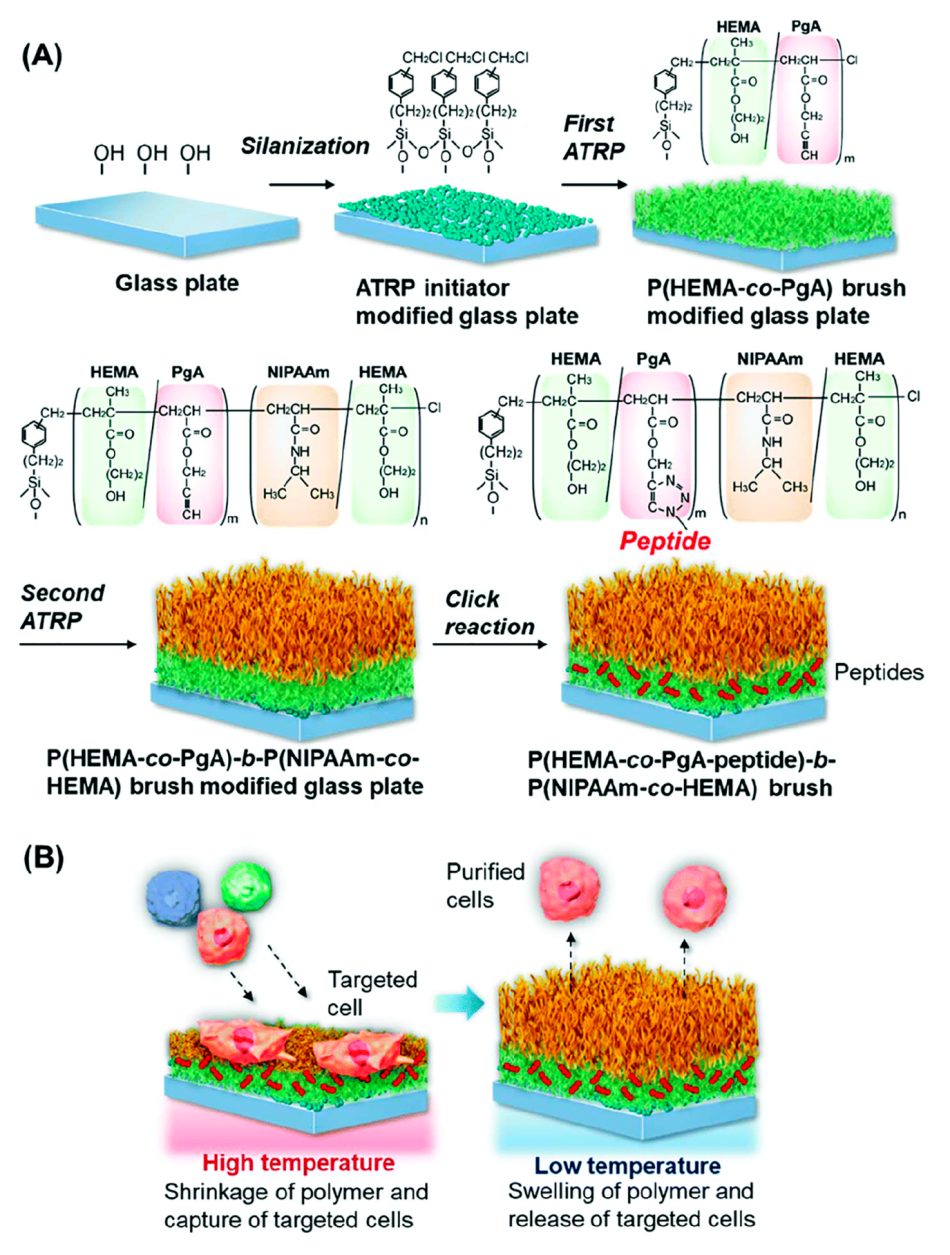
Illustration of the preparation of the grafted surface (A) and temperature-induced cell attachment and release (B). Reprinted with permission from ref (125). Copyright 2021 Royal Society of Chemistry.

The grafting of temperature-responsive PPCs to surfaces has shown great potential in targeting biomolecules by enabling their capture and release. Thermoresponsive RGD-peptide-based polymer conjugates have also been used in the design of responsive surfaces.^122,123^ Cell biorecognition surfaces were developed using poly(*N*-isopropylacrylamide-*co*-acrylamide) P(NIPAm-*co*-Am) as the stimuli-responsive component due to its LCST behavior.^124^ The P(NIPAm-*co*-Am) as well as PEG with a comparable chain length were grafted to a glass surface. To facilitate cell recognition, an integrin binding peptide sequence (GRGDS) was conjugated at the chain end of the P(NIPAm-*co*-Am). The cellular adhesion of the system relies on the interaction between the cellular integrin receptor and the peptide. Temperature was used as an external stimulus to trigger the reversible adhesion and release of the cells. Specifically, increasing the temperature above the LCST of P(NIPAm-*co*-Am) caused the conjugate’s extended chains to switch into a globular structure. Collapse of the conjugate and steric hindrance of the PEG led to the release of the surface-bound cells. Cell adhesion and release systems were also developed using a cyclic RGD peptide (cRGD) conjugated to a thermoresponsive brush copolymer based on NIPAAm, 2-hydroxyethyl methacrylate (HEMA), and propargyl acrylate (PgA) (Figure 15A).^125^ The copolymer containing the cRGD peptide showed fast cell attachment at 37 °C, while the copolymer without the cRGD motif did not exhibit cell adhesion. By an increase in the length of the temperature-responsive segment in the copolymer, the cell attachment and release can be modulated as a function of the temperature. Shrinkage of the thermoresponsive segment at 37 °C was suggested to promote interaction between the cell and the cRGD peptide, leading to cell attachment to the copolymer. Conversely, at 20 °C, the cell is released due to the extended conformation of the solvated and hydrophilic copolymer segment (Figure 15B). Recently, a similar copolymer was grafted onto silica beads and conjugated with exosome affinity peptides to design a temperature-responsive exosome capture system.^126^ Exosomes were also captured and released based on temperature changes caused by the shrinkage or extension of the thermoresponsive copolymer segment.

The use of temperature-responsive PPCs in biomedical applications has been highlighted in this last section. The thermogelation and self-assembly abilities of the presented examples and conjugates enabled the *in vivo* and *in vitro* treatment of wounds, tumors, and delivery of biomolecules. In addition, surface grafting has also been applied to thermoresponsive PPCs for the design of interactive surfaces targeting cellular receptors.

## 6 Conclusions and Future Perspectives

In the design of nanostructured materials, polymer–peptide conjugates stand out from other supramolecular building blocks by combining the benefits of both constituents. The development of stimuli-responsive conjugates enables the capability to control properties, on-demand, and due to their diverse structures and programmable properties, PPCs are of particular interest for the design of adaptive and interactive materials.

This short review provides an overview of temperature-responsive PPCs for the preparation of self-assembled structures in solution, dispersed media, and gels. Pioneering work as well as recent studies were selected to illustrate the chemical routes and design of thermoresponsive PPCs based on ELP, CMP, amyloid peptide, cyclic peptide, polypeptide, and other peptide blocks, as well as various polymers. The temperature-responsive behavior of the PPCs was highlighted by selecting examples that show self-assembly into various supramolecular structures, including 0D nanospheres and nanoparticles, 1D nanotubes and nanofibers, 2D nanosheets and nanoribbons, and 3D networks. The influence of the peptide and/or polymer on the self-assembly and gelation process was discussed as well as potential biomedical applications of temperature-responsive PPCs.

Although some materials have exhibited degradable properties, many studies have been conducted using nondegradable polymers. Current environmental, ecological, pharmacological, and immunological concerns have highlighted the need to introduce more sustainable and degradable materials. By use of degradable polymers, the controlled kinetics of degradation can be adjusted and improved further. In addition, there is a need to diversify the range of applications for PPCs. This challenge can be addressed by gathering knowledge on the synthesis with a better understanding of the structure–property relationship of biomolecules. We have restricted our review and discussion to examples in which temperature-induced transitions are triggered using a stepwise increase or decrease. In the future, more advanced applications might use biological temperature gradients to induce structural changes in thermoresponsive carriers or localized thermogelation processes for accumulation or release profiles.

We believe that this short review will help the scientific community to develop new PPCs, in the development of materials with new functionalities and properties to face current environmental and economic challenges with versatile design, new functions, and targeted properties, to diversify their range of applications.
